# Extracorporeal shock waves protect cardiomyocytes from doxorubicin-induced cardiomyopathy by upregulating survivin via the integrin-ILK-Akt-Sp1/p53 axis

**DOI:** 10.1038/s41598-019-48470-0

**Published:** 2019-08-21

**Authors:** Ji Yoon Lee, Jihwa Chung, Kyoung Hwa Kim, Shung Hyun An, Jeong-Eun Yi, Kyoung Ae Kwon, Kihwan Kwon

**Affiliations:** 1 Medical Research Institute, School of Medicine, Ewha Womans University, Seoul, 158-710 Korea; 2 Department of Internal Medicine, Cardiology Division, School of medicine, Ewha Womans University, Seoul, 158-710 Korea; 3 Graduate School of Industrial Pharmaceutical Sciences, Ewha Womans University, Seoul, Korea

**Keywords:** Cardiomyopathies, Apoptosis

## Abstract

Doxorubicin (DOX) is a widely used anti-cancer drug; however, it has limited application due to cardiotoxicity. Extracorporeal shock waves (ESW) have been suggested to treat inflammatory and ischemic diseases, but the concrete effect of ESW in DOX-induced cardiomyopathy remain obscure. After H9c2 cells were subjected to ESW (0.04 mJ/cm^2^), they were treated with 1 μM DOX. As a result, ESW protected cardiomyocytes from DOX-induced cell death. H9c2 cells treated with DOX downregulated p-Akt and survivin expression, whereas the ESW treatment recovered both, suggesting its anti-apoptotic effect. ESW activated integrin α_v_β_3_ and α_v_β_5_, cardiomyocyte mechanosensors, followed by upregulation of ILK, p-Akt and survivin levels. Further, Sp1 and p53 were determined as key transcriptional factors mediating survivin expression via Akt phosphorylation by ESW. In *in vivo* acute DOX-induced cardiomyopathy model, the echocardiographic results showed that group subjected to ESW recovered from acute DOX-induced cardiomyopathy; left ventricular function was improved. The immunohistochemical staining results showed increased survivin and Bcl2 expression in ESW + DOX group compared to those in the DOX-injected group. In conclusion, non-invasive shockwaves protect cardiomyocytes from DOX-induced cardiomyopathy by upregulating survivin via integrin-ILK-Akt-Sp1/p53 pathway. *In vivo* study proposed ESW as a new kind of specific and safe therapy against acute DOX-induced cardiomyopathy.

## Introduction

Doxorubicin (DOX) is a highly effective anti-cancer agent, particularly when used to treat breast cancer, leukemia and lymphoma^1,2^. However, its long-term clinical use has been limited by cardiotoxicity. Indeed, several studies have demonstrated that the anthracycline DOX induces oxidative stress resulting in apoptosis of cardiomyocytes^1,3^. Consistent with these cellular effects, a cumulative dose of DOX increases the risk of cardiotoxicity with cardiac symptoms similar to those of dilated cardiomyopathy; the cardiac chambers dilate and the ventricular ejection fraction (EF) and contractile function are significantly reduced^4–6^. It is important to identify effective ways to alleviate the side effects of DOX in cancer therapy.

Survivin, a member of the inhibitor of apoptosis protein family, regulates cellular apoptosis and tumor progression in various cell types^7–9^. As DOX treatment induces cellular apoptosis in cardiomyocytes, survivin has been suggested to be an appropriate therapeutic target in patients with DOX-induced cardiomyopathy. Indeed, recent studies have revealed that delivering recombinant survivin to H9c2 cardiomyocytes induces an anti-apoptotic effect against DOX-induced apoptosis^10,11^. However, providing exogenous survivin for therapeutic purposes over the long term is not practical due to its invasiveness and short half-life^12^. Therefore, upregulation of the endogenous survivin level would be a more reasonable potential therapy against DOX-induced cardiomyopathy.

Several studies have suggested that pretreatment with some antioxidant enzymes or insulin confers cardioprotective effects against DOX-induced cardiotoxicity by increasing the survivin level^13–15^. However, these methods have some site-specific effect limitations, where the materials may exert certain adverse effects on cells at other sites^16,17^. In particular, cardioprotective insulin is thought to induce the development and progression of cancer, and anti-cancer drug resistance in cancer cells, which is harmful to patients with cancer^13,18^. Therefore, a new kind of therapy that upregulates site-specific survivin should accompany chemotherapy in patients suffering from cancer.

Extracorporeal shock wave (ESW) therapy has been utilized as a first-line treatment for stone diseases, especially due to its high energy^19,20^. Several studies have demonstrated that low-energy ESW has protective effects in various diseases associated with bones, tendons, and the musculoskeletal system^21^. In particular, ESW promotes angiogenesis and improves cardiac injury after acute myocardial infarction^22^. Our previous study demonstrated that low-energy ESW results in angiogenic gene expression via the mechanosensory complex including VEGFR2, VE-cadherin and PECAM-1 in endothelial cells (ECs)^23^. In addition, ESW treatment activates the PI3K/Akt signaling pathway in both cardiomyocytes and ECs, and is also involved in survivin expression^7,13,23,24^. Based on these results, we hypothesized that ESW upregulates survivin expression through mechanotransduction of cardiomyocytes. Therefore, the present study was carried out to investigate the potential cardioprotective effects of ESW against DOX-induced cardiotoxicty as well as elucidating the concrete mechanism with respect to survivin induction both in *in vitro* and *in vivo* models.

## Results

### ESW stimulates Akt phosphorylation followed by upregulation of survivin expression in cardiomyocytes

**Figure 1:**
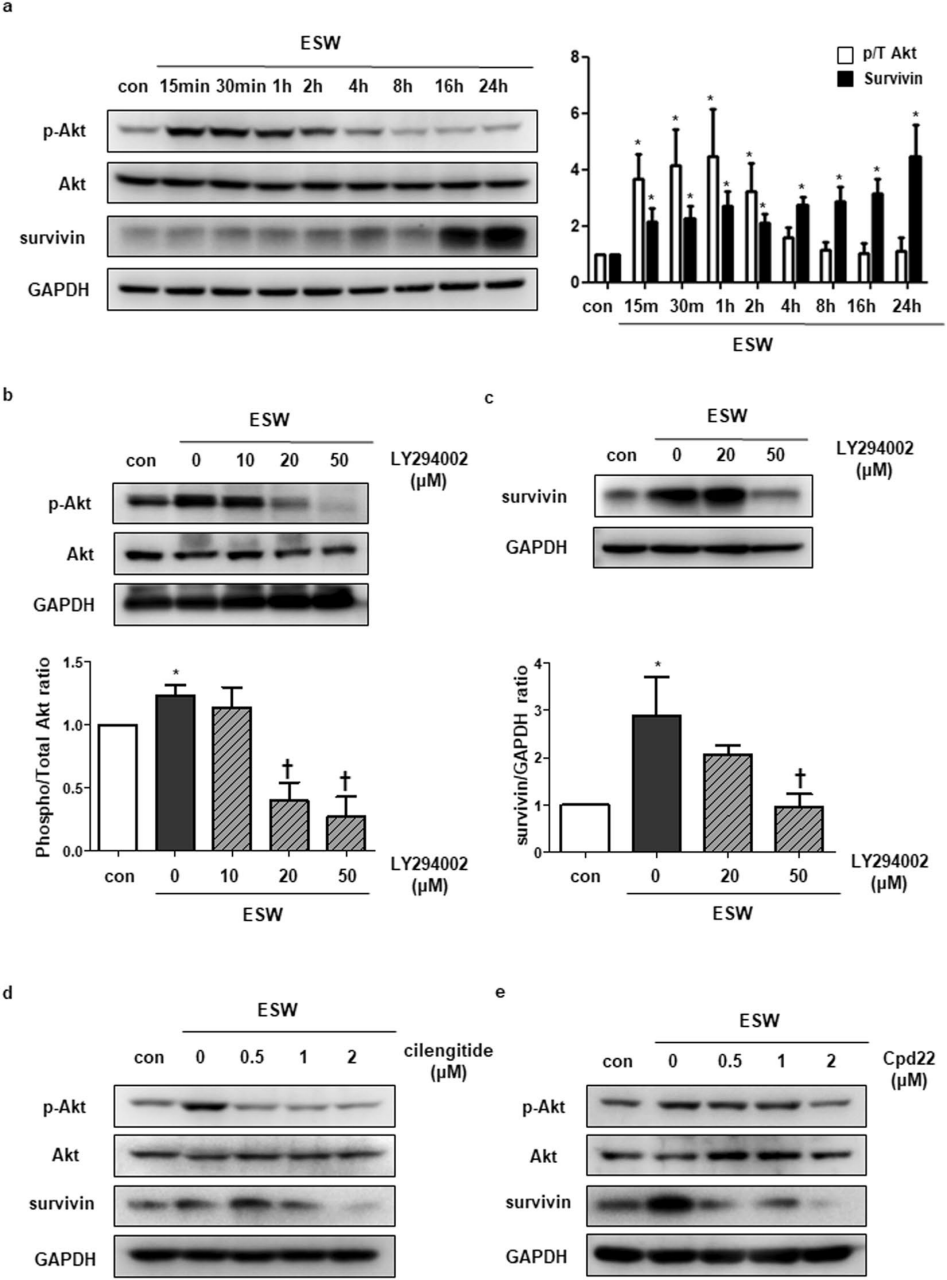
Extracorporeal shock waves (ESW) upregulates survivin through the integrin/ILK/Akt signaling pathway in cardiomyocytes. (**a**) After treatment with 1,000 shots of ESW (0.04 mJ/mm^2^), H9c2 cells were incubated in a 5% CO_2_ incubator at 37 °C for the indicated time periods. The protein levels of p-Akt, Akt, survivin, and GAPDH were measured by Western blot. The protein expression levels were normalized to GAPDH (internal control), and are indicated relative to the control. The full-length blots are presented in Supplementary Fig. S2a. n = 5, Mean ± SEM. *Significant difference compared to control (*p* < 0.05). ^†^Significant difference compared to the DOX condition (*p* < 0.05). (**b**–**d**) and (**e**) The cells were exposed to ESW after treatment with LY294002, cilengitide, or Cpd22 at the indicated concentrations for 2 h or left untreated. The cells were harvested after a 30 min or 24 h incubation under a static condition. The protein expression levels of p-Akt, Akt, survivin, and GAPDH were measured by Western blot and normalized to GAPDH (internal control), and are indicated relative to those of the control. Representative images from at least three independent results are shown. The full-length blots are presented in Supplementary Fig. S2b,c, S3a,c. *Significant difference compared to control (*p* < 0.05). ^†^Significant difference compared to the DOX condition (*p* < 0.05).

H9c2 cells were treated with ESW and incubated for the indicated time periods to investigate whether Akt phosphorylation is regulated by ESW stimulation in cardiomyocytes. As shown in Fig. 1a, ESW upregulated phosphorylation of Akt, while total Akt expression remained constant; the highest p-Akt level was observed at 30 min after the ESW treatment. Cells exposed to ESW showed increased *survivin* mRNA expression compared to control (Supplementary Fig. S1a). Survivin protein levels also increased significantly in cells at 16 h after being exposed to ESW (Fig. 1a). The upregulated expression of survivin was maintained until 24 h after the ESW treatment. To clarify whether ESW regulates Akt phosphorylation followed by upregulation of survivin, we inhibited Akt phosphorylation using the PI3K inhibitor LY294002. As shown in Fig. 1b, 50 μM of LY294002 significantly suppressed the increased level of Akt phosphorylation by ESW in H9c2 cells. Furthermore, treatment with a PI3K inhibitor at this concentration significantly downregulated the elevated expression of survivin induced by ESW, suggesting that phosphorylation of Akt by ESW induces survivin expression in cardiomyocytes (Fig. 1c).

### Integrin as a mechanosensor of cardiomyocytes regulates the Akt/survivin axis by ESW

To determine whether survivin expression is regulated by activation of the mechanosensory complex that senses stretch by ESW, we tested two mechanosensor candidates related to Akt phosphorylation in cardiomyocytes, namely AT1R and integrin (α_v_β_3_ and α_v_β_5_)^25–27^. H9c2 cells were treated with losartan potassium or cilengitide, which are AT1R and integrin (α_v_β_3_ and α_v_β_5_) inhibitors, respectively. After 2 h of chemical treatment, the cells were exposed to ESW followed by incubation for the indicated time periods. Inhibiting the two kinds of mechanosensors showed a deficiency in delivering the upstream signaling pathway in response to ESW; the elevated p-Akt levels caused by ESW decreased. Furthermore, inhibiting integrin suppressed upregulated survivin expression caused by ESW, while inhibiting AT1R had no effect on survivin expression (Fig. 1d, Supplementary Fig. S1b,c, and Supplementary Fig. S4). These results indicate that integrin acts as a key mechanosensor regulating survivin expression by ESW in cardiomyocytes. To clarify whether activation of integrin affected the Akt/survivin axis, ILK was inhibited, which blocks downstream integrin activation^28^. As a result, cells treated with the ILK inhibitor Cpd22 showed decreased levels of both p-Akt and survivin, which were upregulated by ESW (Fig. 1e and Supplementary Fig. S1c). These results indicate that integrin initiates mechanotransduction by ESW in cardiomyocytes followed by activation of ILK, which regulates the Akt/survivin axis.

### ESW protects cardiomyocytes from DOX-induced cell death

**Figure 2:**
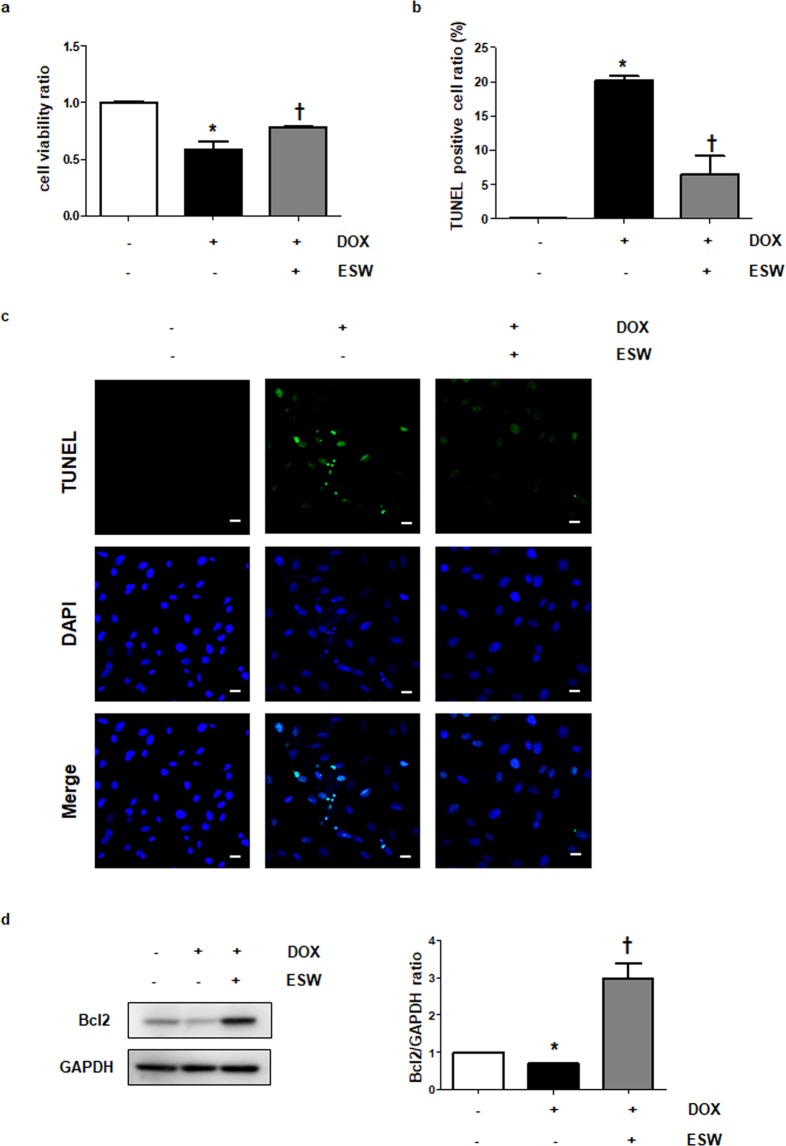
ESW protects cardiomyocytes from doxorubicin (DOX)-induced cell death. H9c2 cells were left untreated or treated with 1,000 shots of ESW (0.04 mJ/mm^2^), and then statically incubated in a 5% CO_2_ incubator for 1 h. After the incubation, cells were treated with 1 μM DOX for 24 h. (**a**) Cell viability under each condition was measured by the MTT assay. The values of all conditions are indicated relative to the control. n = 5, Mean ± SEM. *Significant difference compared to control (*p* < 0.05). ^†^Significant difference compared to the DOX condition (*p* < 0.05). (**b**) The bar graph shows the terminal deoxynucleotidyl transferase dUTP nick-end labeling (TUNEL)-positive cell ratio (%) as a cellular apoptotic index. The TUNEL-stained cells (green) were counted and normalized by the DAPI-stained cells (blue): control (0.08833 ± 0.009458, means ± standard error), DOX (20.17 ± 0.6499, means ± standard error) and ESW + DOX (6.516 ± 2.644, means ± standard error). n = 5, Mean ± SEM. *Significant difference compared to control (*p* < 0.05). ^†^Significant difference compared to DOX condition (*p* < 0.05). (**c**) Representative fluorescence microscopic TUNEL images from at least three independent experiments are shown (magnification, 200×; scale bars, 10 μm). (**d**) Bcl2 protein expression was measured by Western blotting. The protein expression levels were normalized to GAPDH (internal control), and are indicated relative to those of the control. The full-length blots are presented in Supplementary Fig. S6. n = 5, Mean ± SEM. *Significant difference compared to control (*p* < 0.05). ^†^Significant difference compared to DOX condition (*p* < 0.05).

As revealed previously^12^, treatment with 1 μM DOX for 24 h reduced H9c2 cell viability. However, as shown in Fig. 2a, stimulation with ESW prior to the DOX treatment significantly alleviated the reduced cell viability. The DOX treatment induced greater cellular apoptosis in the TUNEL assay compared to the control, whereas pretreatment with ESW suppressed apoptosis (Fig. 2b,c). Furthermore, we measured the expression of Bcl2, a representative anti-apoptotic protein. As shown in Fig. 2d, Bcl2 protein expression was lower in the DOX-treated condition than in the control. However, ESW stimulation prior to the DOX treatment enhanced the downregulated Bcl2 expression by DOX. These results indicate that ESW protects cardiomyocytes against DOX-induced cellular apoptosis.

### ESW inhibits DOX-induced downregulation of survivin via the integrin/ILK/Akt pathway in cardiomyocytes

**Figure 3:**
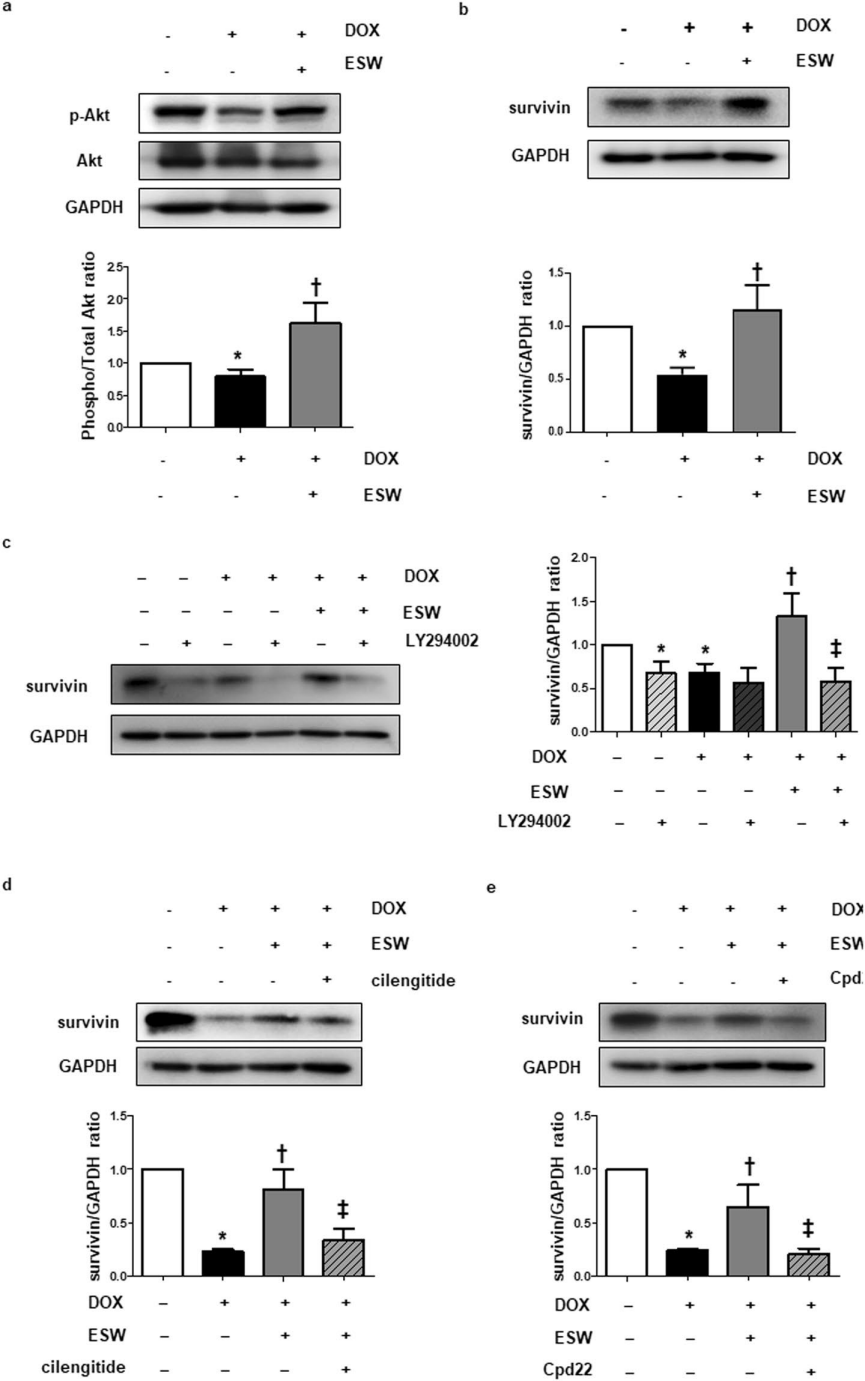
ESW inhibits DOX-induced downregulation of survivin via the integrin/ILK/Akt pathway in cardiomyocytes. (**a**,**b**) H9c2 cells were cultured in 1 μM DOX for 8 or 24 h 1 h after ESW. The protein expression levels of p-Akt, Akt, survivin, and GAPDH were measured by Western blot and normalized to GAPDH (internal control). The expression levels are indicated relative to those of the control. The full-length blots are presented in Supplementary Fig. S7a,b. (**c**) After treatment with 50 μM LY294002 for 2 h, the cells were exposed to ESW or left unexposed. After a 1 h incubation, the cells were treated with 1 μM DOX for 24 h. The full-length blots are presented in Supplementary Fig. S7c. (**d**,**e**) After treatment with 2 μM cilengitide or Cpd22 for 2 h, the cells were exposed to ESW or left unexposed. After a 1 h incubation, the cells were cultured in 1 μM DOX for 24 h. The protein expression levels of survivin and GAPDH were measured by Western blot and normalized to GAPDH (internal control). The expression levels are indicated relative to the control. The full-length blots are presented in Supplementary Fig. S8. n = 5, Mean ± SEM. *Significant difference compared to control (*p* < 0.05). ^†^Significant difference compared to the DOX condition (*p* < 0.05). ^‡^Significant difference compared to the ESW + DOX condition (*p* < 0.05).

Consistent with previous studies^10,12,13^, phosphorylation of Akt, survivin and Bcl2 protein levels were significantly downregulated in H9c2 cells treated with 1 μM DOX for 24 h (Supplementary Fig. S5). We hypothesized that ESW increased the downregulated survivin expression by DOX via the integrin/ILK/Akt/survivin pathway. H9c2 cells were stimulated with ESW 1 h prior to the DOX treatment for the indicated time periods to determine if ESW could recover the survivin level by phosphorylating Akt under the DOX-treated condition. As shown in Fig. 3a,b, DOX treatment suppressed both p-Akt and survivin levels in cardiomyocytes, while subjecting ESW to cells prior to DOX treatment recovered these levels. To determine whether Akt phosphorylation and activating integrin/ILK by ESW also affected survivin expression in the DOX-treated condition, the cells were treated with the inhibitors of PI3K,integrin and ILK, respectively. The results showed that inhibiting Akt phosphorylation with LY294002 attenuated the ESW-induced survivin level in DOX-treated H9c2 cells (Fig. 3c). Similarly, inhibiting integrin and ILK decreased both p-Akt and survivin, which were recovered by ESW under the DOX-treated condition, suggesting a deficiency of cells responding to ESW (Fig. 3d,e). These results indicate that cells treated with an additional ESW treatment recovered their survivin levels suppressed by DOX and the integrin/ILK/Akt/survivin pathway is involved in this mechanism.

### Knockdown of survivin attenuates the anti-apoptotic effect of ESW in DOX-induced cell death

**Figure 4:**
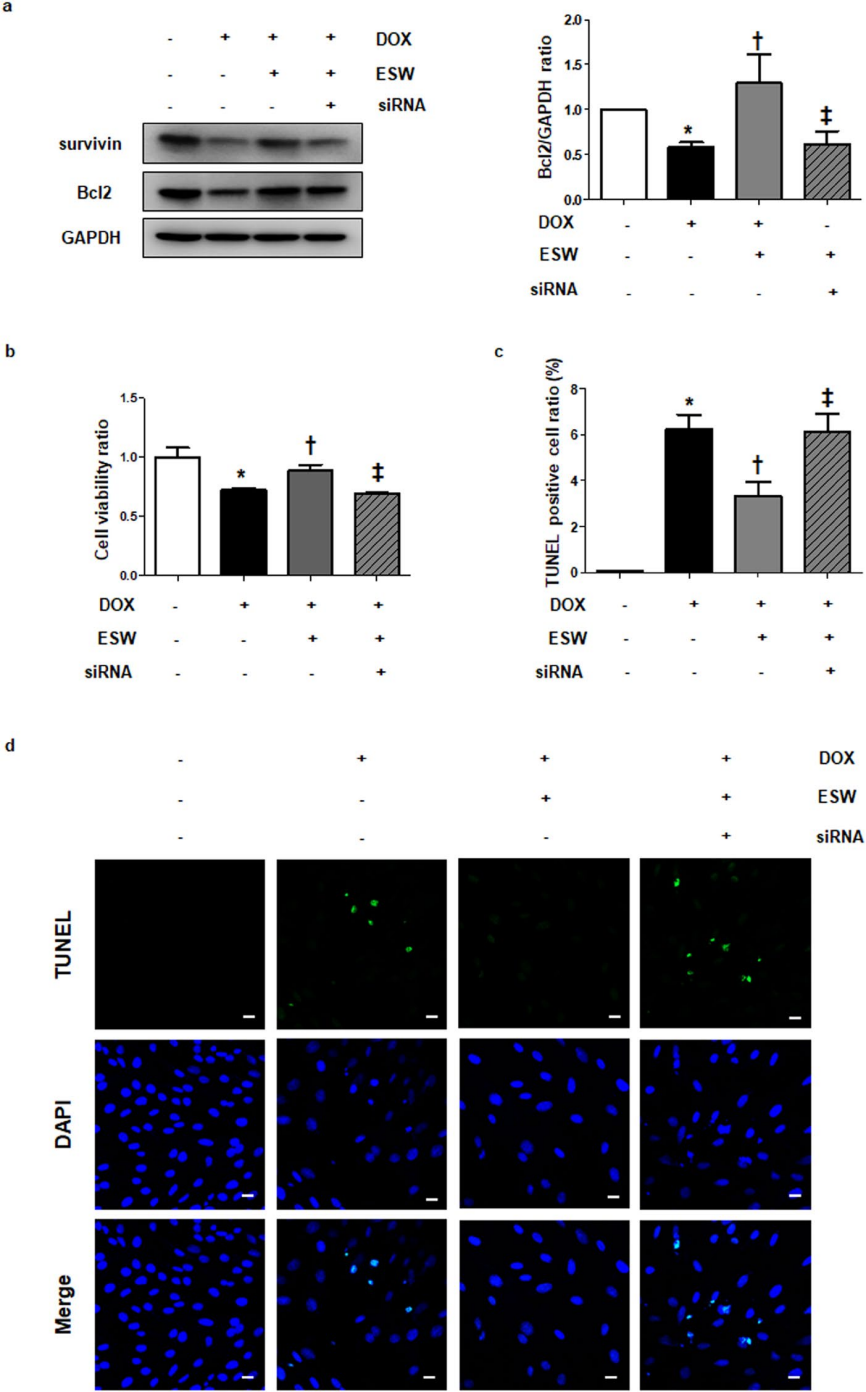
Knockdown of survivin alleviates the cardioprotective effect of ESW in DOX-induced cell death. After transfection with 50 nM siRNA targeting *survivin* for 24 h, H9c2 cells were subjected to ESW 1 h prior to the DOX treatment. The cells were harvested after a 24 h incubation. (**a**) The protein expression levels of survivin, Bcl2, and GAPDH were measured by Western blot and normalized to GAPDH (internal control). The expression levels are indicated relative to those of the control. The full-length blots are presented in Supplementary Fig. S10. (**b**) Cell viability of each condition was measured by the MTT assay. The values for all conditions are indicated relative to those of the control. (**c**) The bar graph shows the terminal deoxynucleotidyl transferase dUTP nick-end labeling (TUNEL)-positive cell ratio (%) as a cellular apoptotic index. The TUNEL-stained cells (green) were counted and normalized by the DAPI-stained cells (blue): control (0.05893 ± 0.03628, means ± standard error), DOX (6.212 ± 0.6617, means ± standard error) and ESW + DOX (3.331 ± 0.6028, means ± standard error), siRNA + ESW + DOX (6.149 ± 0.7767, means ± standard error). n = 5, Mean ± SEM. *Significant difference compared to control (*p* < 0.05). ^†^Significant difference compared to the DOX condition (*p* < 0.05). ^‡^Significant difference compared to the ESW + DOX condition (*p* < 0.05). (**d**) Representative fluorescence microscopic TUNEL images from at least three independent experiments are shown (magnification, 200×; scale bars, 10 μm).

To determine whether survivin is the major factor contributing to the anti-apoptotic effect of ESW in DOX-induced cardiotoxicity, the effect of ESW on DOX-treated cardiomyocytes was observed in which the survivin level was suppressed by siRNA transfection. As shown in Supplementary Fig. S9a, transfection of siRNA targeting *survivin* effectively reduced the protein level of survivin. After 24 h of siRNA transfection, H9c2 cells were subjected to ESW 1 h prior to a 24 h of DOX treatment. When the survivin level was suppressed by siRNA, the level of recovered anti-apoptotic Bcl2 expression by ESW in DOX-treated cells decreased significantly (Fig. 4a and Supplementary Fig. S9b). We also measured cell viability and apoptosis by the MTT and TUNEL assays, respectively. The results showed that knockdown of survivin reversed the effect of ESW in DOX-induced apoptosis; cell viability decreased, while cellular apoptosis increased (Fig. 4b–d). Similarly, inhibition of survivin by the potent survivin inhibitor YM155^29–31^ also attenuated anti-apoptotic effect of ESW in DOX-treated cardiomyocytes (Supplementary Fig. S11, S12). These results show that survivin plays a key role in the anti-apoptotic effect of ESW against DOX-induced death of cardiomyocytes.

### Phosphorylation of Akt by ESW induces survivin by regulating p53 and Sp1 in DOX-treated cardiomyocytes

**Figure 5:**
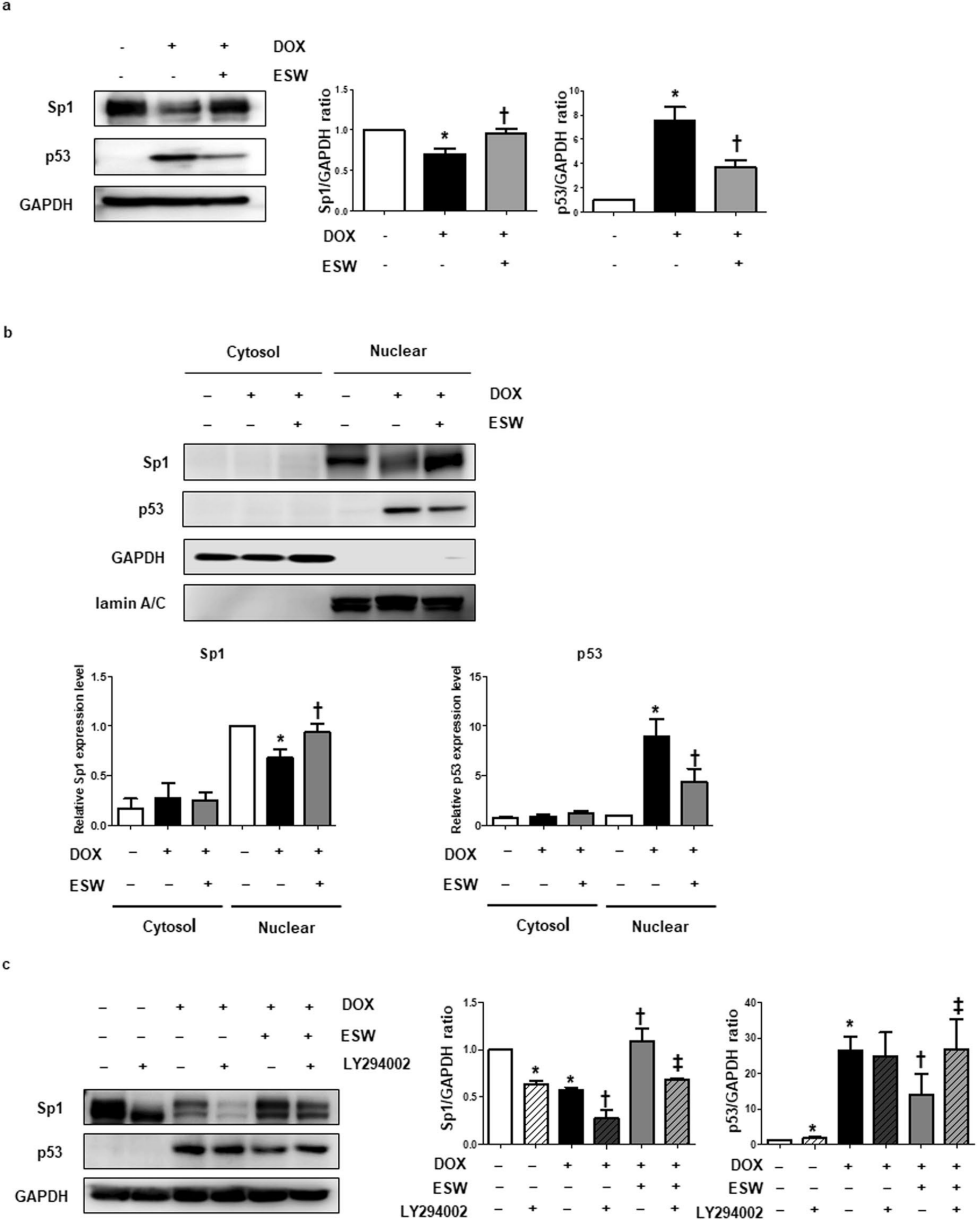
Phosphorylation of Akt by ESW induces survivin by downregulating p53 and upregulating Sp1. The cells were cultured in 1 μM DOX for 24 h after being subjected or not to ESW. (**a**) The protein expression levels of Sp1, p53, and GAPDH were measured by Western blot and normalized to GAPDH (internal control). The expression levels are indicated relative to those of the control. The full-length blots are presented in Supplementary Fig. S14a. (**b**) Cytoplasmic and nuclear extracts isolated from H9c2 cells under each condition were separated by Western blot with antibodies against Sp1, p53, GAPDH, and lamin A/C and are indicated relative to those of the control. GAPDH and lamin A/C were used as the loading controls for the cytosolic and nuclear fractions, respectively. The full-length blots are presented in Supplementary Fig. S14b. (**c**) After treatment with 50 μM LY294002 for 2 h, the cells were exposed to ESW or left unexposed. After a 1 h incubation, the cells were treated with 1 μM DOX for 24 h. The full-length blots are presented in Supplementary Fig. S15. n = 5, Mean ± SEM. *Significant difference compared to the control (*p* < 0.05). ^†^Significant difference compared to the DOX condition (*p* < 0.05). ^‡^Significant difference compared to the ESW + DOX condition (*p* < 0.05).

To identify the key transcription factors mediating survivin expression by ESW, we tested Sp1 and p53, which are known to regulate survivin expression under various stimuli^13^. p53 expression is induced by DOX treatment in cardiomyocytes, whereas DOX inhibits Sp1 activation^13,32,33^. Based on these studies, phosphorylation of Akt by ESW is thought to regulate both Sp1 and p53, and induce survivin expression. Consistent with previous studies, the expression levels of both Sp1 and p53 changed in response to DOX treatment of H9c2 cells; Sp1 level was downregulated, and the p53 level increased following DOX treatment (Supplementary Fig. S13). Furthermore, we investigated whether ESW treatment regulates activation of Sp1 and p53 in cardiomyocytes to a greater extent compared to DOX-only treated cells. As shown in Fig. 5a, cells subjected to ESW reversed the activities of both Sp1 and p53 induced by DOX treatment in H9c2 cells; downregulated Sp1 by DOX increased significantly in cells exposed to additional ESW, while upregulated p53 in response to DOX decreased. Cytosol/nuclear fractionation was performed to determine whether nuclear activation of these two transcription factors is regulated by ESW under DOX-treated conditions. The downregulated nuclear translocation of Sp1 by DOX was recovered in cells subjected to additional ESW. Conversely, increased nuclear translocation of p53 by DOX decreased after the additional ESW treatment (Fig. 5b). Next, it was determined whether regulation of both Sp1 and p53 by ESW was mediated by Akt phosphorylation. After treatment with or without LY294002 for 2 h, the cells were subjected to ESW 1 h prior to the DOX treatment. After a 24 h incubation, we compared the activation of Sp1 and p53 between the control and LY294002-treated groups. As expected, the activities of Sp1 and p53 were reversed in cells treated with additional ESW compared to DOX-treated cells. However, the ESW effects were alleviated in DOX-treated cells when Akt phosphorylation was inhibited by LY294002 (Fig. 5c). These results suggest that phosphorylation of Akt by ESW induces Sp1 and inhibits p53 activation, followed by upregulation of survivin expression in DOX-treated cardiomyocytes.

### ESW improves acute DOX-induced cardiomyopathy, while inhibition of survivin reverses the cardioprotective effect of ESW in an *in vivo* mouse model

**Figure 6:**
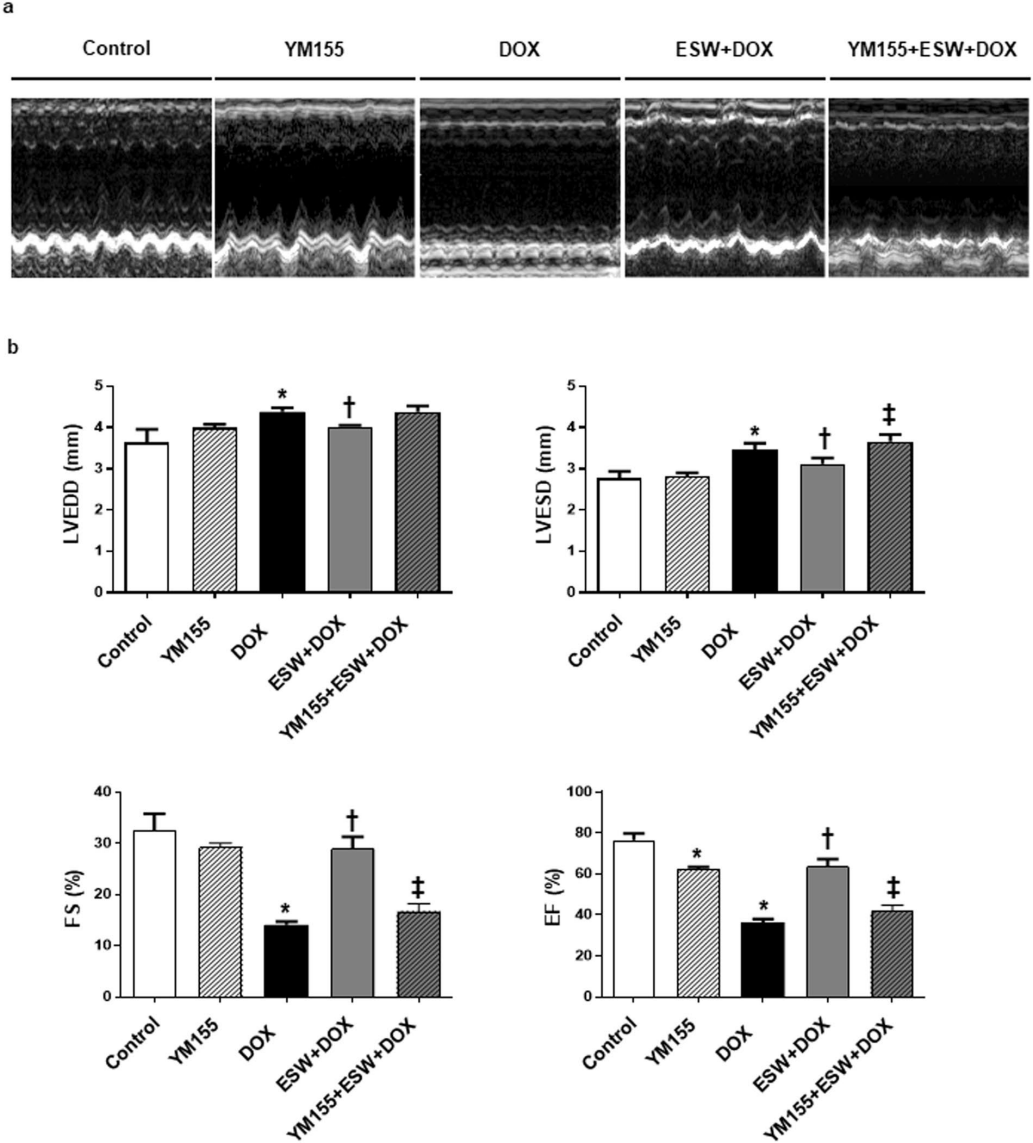
ESW improves acute DOX-induced cardiomyopathy, while inhibition of survivin reverses the cardioprotective effect of ESW in an *in vivo* mouse model. (**a**) Representative M-mode images from the control, YM155, DOX ESW + DOX, and YM155 + ESW + DOX groups. (**b**) Quantitative echocardiographic group data: LVEDD (mm), LVESD (mm), FS (%) and EF (%). n = 8/group, Mean ± SEM. *Significant difference compared to control (*p* < 0.05). ^†^Significant difference compared to the DOX condition (*p* < 0.05). ^‡^Significant difference compared to the ESW + DOX group (*p* < 0.05).

**Figure 7:**
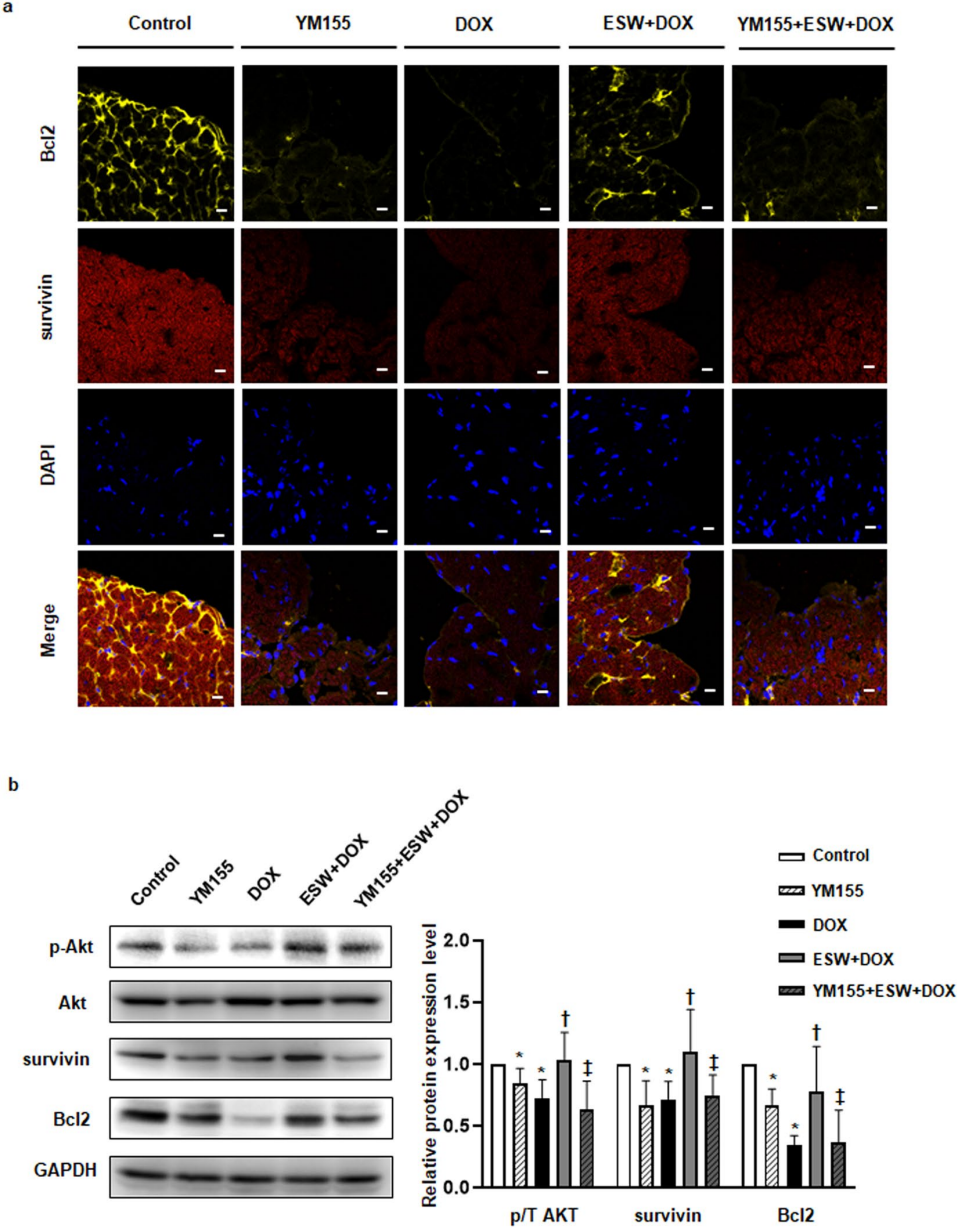
ESW attenuates cardiomyocyte apoptosis by upregulation of phosphorylation of Akt and survivin followed by upregulation of Bcl2 in an *in vivo* mouse model. (**a**) Immunofluorescence staining to detect Bcl2 and survivin in various regions of the mice myocardium. After fixation, the tissues were stained with anti-Bcl2 antibody (yellow) and anti-survivin antibody (red), while nuclei were stained with DAPI (blue). The levels of Bcl2 and survivin expression were compared among the three groups (control, YM155, DOX, ESW + DOX, and YM155 + ESW + DOX) using confocal microscopy. Representative images are shown (magnification 400×; scale bars, 20 μm). (**b**) The hearts were harvested at 14 days after the DOX injection and examined by Western blot for p-Akt, Akt, survivin, Bcl2, and GAPDH. The expression levels were normalized to GAPDH (internal control) and are indicated relative to those of the control (n = 8/group). Representative images from at least three independent results are shown. *Significant difference compared to control (*p* < 0.05). ^†^Significant difference compared to the DOX condition (*p* < 0.05). ^‡^Significant difference compared to the ESW + DOX group (*p* < 0.05). The English in this document has been checked by at least two professional editors, both native speakers of English. For a certificate, please see: http://www.textcheck.com/certificate/ycCcrH.

To explore the effects of ESW on acute DOX-induced cardiomyopathy *in vivo*, cardiac functions and body weights among the control, YM155, DOX, ESW + DOX, and YM155 + ESW + DOX groups were compared (Fig. 6a,b and Supplementary Fig. S16). The DOX group had significantly dilated LV chamber dimensions, lower FS and EF and lower body weight compared to the control group, suggesting DOX-induced cardiomyopathy^34,35^. However, the ESW + DOX group showed significantly improved LV function, as well as reduced LV chamber dimensions compared to the DOX group, which was similar to the control group (Supplementary Table S1, Fig. 6a,b). As shown in Supplemental Table S1, the ESW + DOX group recovered both LV FS (%) and EF (%), which were decreased by DOX from 14.08 ± 0.6575 to 28.98 ± 2.292 (*p* = 0.0079) and from 36.48 ± 1.452 to 63.85 ± 3.468 (*p* = 0.0079), respectively. Moreover, cardiac functions of ESW + DOX and YM155 + ESW + DOX groups were compared to identify whether survivin induced by ESW is the crucial factor for its cardioprotective effect. As shown in Fig. 7a,b, it was confirmed that YM155 injection significantly attenuated survivin expression on cardiomyocytes; mice which received YM155 injections showed significantly lower levels of survivin than control. With lower levels of survivin, YM155 + ESW + DOX group showed decreased body weight and LV function with dilated LV chamber dimensions compared to ESW + DOX group (Supplementary Fig. S16, Supplementary Table S1 and Fig. 6a,b). As shown in Supplemental Table S1, the YM155 + ESW + DOX group showed worse LV FS (%) and EF (%) compared to ESW + DOX group from 28.98 ± 2.292 to 16.74 ± 1.432 (*p* = 0.0014) and from 63.85 ± 3.468 to 41.94 ± 2.734 (*p* = 0.0014), respectively. From these results, it was confirmed that ESW improves cardiac functions in DOX-induced cardiomyopathy and survivin plays a key role in its cardioprotective effect.

### ESW has anti-apoptotic effects on DOX-induced cardiotoxicity in an *in vivo* mouse model by upregulating Akt phosphorylation, survivin and Bcl2 expression

To investigate whether survivin induced by ESW indeed contributes to anti-apoptotic effect on myocardium in the acute DOX-induced cardiomyopathy model, the expression levels of Bcl2, the anti-apoptotic protein, were compared among control, YM155, DOX, ESW + DOX, and YM155 + ESW + DOX groups. Both the immunohistochemical staining and Western blot results showed that the expression levels of Bcl2 were downregulated in DOX group compared to control group, while ESW + DOX group showed elevated Bcl2 expression level compared to DOX group. Furthermore, recovered Bcl2 expression level in ESW + DOX group was downregulated in the YM155 + ESW + DOX group (Fig. 7a,b and Supplementary Fig. S17).

To identify the involvement of Akt/survivin axis in response to ESW on myocardium in the acute DOX-induced cardiomyopathy model, we evaluated the levels of Akt phosphorylation and survivin in heart tissues of the control, YM155, DOX, ESW + DOX, and YM155 + ESW + DOX groups by immunohistochemical staining or Western blot. The results showed that the expression levels of both were decreased in the DOX group compared to the control group, but were recovered in the ESW + DOX group. However, YM155 + ESW + DOX group showed suppressed level of both Akt phosphorylation and survivin compared to ESW + DOX group (Fig. 7a,b and Supplementary Fig. S17). These results suggest that Akt/survivin pathway is involved in the anti-apoptotic effect of ESW against DOX-induced cardiotoxicity followed by upregulating Bcl2 expression. Similar with *in vitro* results, it was concluded that survivin exerts the major role in anti-apoptotic effect of ESW against DOX-induced cardiotoxicity by attenuating apoptosis of myocardium followed by improving cardiac functions.

## Discussion

Our study confirmed that ESW induces survivin expression in cardiomyocytes, which improved DOX-induced cardiotoxicity. Furthermore, we demonstrated that mechanotransduction of the integrin-ILK-Akt-Sp1/p53 pathway is the key mechanism underlying the regulation of survivin expression by ESW.

Previous studies have demonstrated that ESW is an effective therapy for acute myocardial infarction and refractory angina pectoris due to its potential angiogenic effects^22^. In other studies, ESW upregulated angiogenic factors, such as p-Akt, p-Erk, endothelial nitric oxide synthase, and vascular endothelial growth factor in HUVECs, which contribute to recirculating the blood during myocardial infarction and ischemia^23,36^. Similarly, ESW plays a therapeutic role by alleviating cellular apoptosis^24^. However, the concrete mechanism of how ESW alleviates cellular apoptosis in cardiomyopathy remains unknown.

Phosphorylation of Akt induced by ESW is suggested to be an important signal change contributing to its therapeutic effect in various cell types^10,23,24^. In addition, survivin is a crucial factor that inhibits cellular apoptosis in cardiomyocytes^13,15^. We hypothesized that the anti-apoptotic survivin level increased in response to ESW, and that its level was regulated by ESW-induced Akt phosphorylation. The results of this study show that the ESW treatment induced Akt phosphorylation within 1 h and then upregulated survivin expression from 16 to 24 h. Furthermore, inhibiting p-Akt with an inhibitor suppressed upregulation of survivin by ESW. From these results, we clarified that phosphorylation of Akt induced by ESW upregulates survivin expression in cardiomyocytes.

To investigate whether ESW has anti-apoptotic effects in DOX-induced cardiotoxicity *in vitro*, an *in vitro* experiment was established with the ESW + DOX group. Based on our preliminary experiments, we chose a 0.04 mJ/mm^2^ energy level and 1000 shots as ESW, as this condition induces both phosphorylation of Akt and survivin expression most effectively in cardiomyocytes (Supplementary Fig. S18). The cells for the *in vitro* ESW + DOX group were subjected to ESW 1 h before the DOX treatment. Upregulation of p-Akt by ESW before DOX administration would be appropriate to examine the effect of ESW on DOX-induced cell death. ESW treatment in advance of anti-cancer chemotherapy is thought to be reasonable. Under these *in vitro* conditions, additional ESW treatment in DOX-treated cardiomyocytes showed anti-apoptotic effects; both cell viability and cellular apoptosis improved and the anti-apoptotic Bcl2 level was recovered compared to those in the DOX group.

Previous studies have demonstrated that various chemical or mechanical stimuli are involved in mechanotransduction of cardiomyocytes. Mechanosensors allow cells to respond to distinct shear stress and convert mechanical forces into biological signals^37,38^. In particular, stretch is a major mechanical force that activates myocardial signaling. Various cellular components have been proposed as types of mechanosensors, such as ion channels, G-protein coupled receptors, AT1R, and integrin^25,26^. Among them, AT1R and integrin are best known for their ability to phosphorylate Akt in response to myocardial stretch^25,27,39^. Because ESW activates phosphorylation of Akt in cardiomyocytes, AT1R and integrin would be key mechanosensors that transfer mechanical force into cellular signaling pathways in response to ESW. Integrins are associated with cytoskeletal and signaling proteins and form a critical mechanotransducer carrying bidirectional signaling pathways^40^. Although it is known that β_1_ integrin expression is dominant, both β_3_ and β_5_-integrins have been reported in cardiomyocytes^40–44^. Furthermore, integrin α_v_β_3_- and α_v_β_5_-induced activation of Akt occurs in response to ligand binding and exerts an anti-apoptotic effect under H_2_O_2_-treated conditions in cardiomyocytes^27,45^. Based on these results, we chose integrins α_v_β_3_ and α_v_β_5_ as candidate cardiomyocyte mechanosensors and confirmed that they transferred mechanical forces of the ESW into cellular signaling in cardiomyocytes. However, inhibiting AT1R had few effects on the survivin expression induced by ESW under our *in vitro* ESW experimental settings. Although angiotensin II stimulates survivin expression in ECs, a complex relationship exists between specific stimuli and the corresponding mechanosensors involved in survivin expression in various cell types^46,47^. Our data show that the integrin inhibitor decreased the Akt phosphorylation induced by ESW followed by suppression of survivin expression. These results suggest that integrin is an effective cardiomyocyte mechanosensor in response to ESW.

ILK is highly expressed in cardiac muscle, where it plays a critical role in cell migration and development of cardiac diseases related to integrin function. Bound to integrin, ILK links integrins and receptor tyrosine kinases to the actin cytoskeleton and downstream signaling molecules, particularly to activate Akt^28^. ILK is involved in arteriogenesis of the heart by ESW^22^. In addition, a previous study showed that conditional deletion of ILK in mouse cardiomyocytes results in the development of dilated cardiomyopathy^48^. Based on these results, ILK is thought be activated by ESW via integrin, which would in turn induce Akt phosphorylation. Indeed, inhibiting ILK downregulated the p-Akt and survivin expression induced by ESW. Furthermore, recovery of survivin by ESW under the DOX-treated condition was attenuated by inhibiting ILK. Therefore, the anti-apoptotic effect of ESW in DOX-induced cardiotoxicity was mediated by the integrin-ILK-Akt-survivin signaling pathway.

Transcription of survivin in cardiomyocytes is regulated by several transcription factors. However, it is unknown which transcription factors directly regulate survivin expression under the ESW treatment condition. To test the key transcription factors regulating survivin expression induced by ESW in cardiomyocytes, Sp1 and p53 were chosen as candidates based on previous data. As expected, the DOX treatment downregulated Sp1 activation and upregulated p53 activation compared to the control. However, the ESW + DOX treatment reversed this; the downregulated Sp1 level was recovered and the upregulated p53 level was ameliorated. Next, we investigated whether phosphorylation of Akt by ESW directly regulates Sp1 and p53 transcriptional activity. Inhibiting Akt phosphorylation attenuated the regulatory ability of ESW on those two transcription factors under the DOX-treated condition. Thus, Sp1 and p53 are suggested as key transcription factors involved in regulating survivin expression induced by ESW under the DOX-treated condition in cardiomyocytes.

Finally, we demonstrated that consistent ESW + DOX administration improved cardiac systolic function compared to that in the DOX-only treated group. For the *in vivo* study, the short-term model with single injection of DOX (15 mg/kg, i.p.)^35,49,50^, instead of long-term model with consecutive injection at lower dose^51^, was used to avoid prolonged exposure to inhalational anesthesia during ESW treatment and repetitive interaction between DOX and anesthesia. Both the immunohistochemical staining and Western blot results suggested that the DOX group had lower expression of p-Akt, survivin and Bcl2 than the control group. However, the ESW + DOX group showed significantly higher levels of those proteins compared to the DOX group, suggesting an anti-apoptotic effect of ESW in DOX-induced cardiomyopathy. Further, inhibition of survivin using YM155 reversed the cardioprotective effect of ESW on acute DOX-induced cardiomyopathy *in vivo* model. Thus, ESW has a clinical therapeutic effect on acute DOX-induced cardiomyopathy in mouse by upregulating survivin expression.

In conclusion, this study demonstrated the mechanism by which ESW protects cardiomyocytes from acute DOX-induced cardiomyopathy. In addition, the relationship between ESW and survivin expression in cardiomyocytes was clarified, and the integrin-ILK-Akt pathway (via mechanotransduction) is involved in this mechanism. In particular, we propose an anti-apoptotic effect of ESW based on *in vivo* evidence that clinical use of ESW in mice with DOX-induced cardiomyopathy improved cardiac function. A new perspective of ESW is suggested as a cardiac lesion-specific therapy for patients with cancer receiving anthracycline treatment to induce survivin expression via a mechanotransduction-mediated signaling pathway.

## Methods

### Cell culture and reagents

The rat neonatal H9c2 cardiac myoblast cell line was obtained from the Korea Cell Line Bank (Seoul, Korea). The cells were cultured in Dulbecco’s modified Eagles medium (DMEM)/high glucose with 10% fetal bovine serum and 1% penicillin-streptomycin (Corning/Mediatech, Inc., Manassas, VA, USA,).

DOX was purchased from Tocris Bioscience (Cat#2252, Bristol, UK). LY294002 (Cat#1130), the PI3K inhibitors, cilengitide (Cat#5870), and losartan potassium (Cat#3798), the integrin inhibitors α_v_β_3_ and α_v_β_5_, and the angiotensin II type 1 receptor (AT1R) inhibitors respectively, were purchased from Tocris Bioscience. The ILK inhibitor Cpd22 (Cat#407331) was obtained from EMD Millipore Corp. (Darmstadt, Germany). The survivin inhibitor YM-155 was purchased from MedChem Express (Cat#HY-10194, Monmouth Junction, NJ, USA).

### *In vitro* ESW experiments

Confluent H9c2 cells were cultured in 40-mm dishes and exposed to ESW using Regenwave ESWT^TM^ (HNT Medical, Seoul, Korea). As described previously, the cells were directly subjected to ESW treatment by perpendicularly immersing a sterile ESW probe into the plate and positioning it just enface to the surface of the H9c2 medium^23^. H9c2 cells were exposed to 1,000 shots of ESW at 4 Hz and then incubated in a 5% CO_2_ incubator.

### *In vivo* experiments in animal model of DOX-induced cardiomyopathy

Animal studies were performed according to the Guidelines for Animal Experiments and were approved by the Animal Experimentation Ethics Committee of Ewha Womans University. All animal procedures were conducted under inhalational anesthesia with 0.5 L/min of oxygen and 1–2% Terrell isoflurane (Minrad International Inc., Orchard Park, NY, USA). Male C57BL/6 mice (Central Animal Laboratory, Seoul, Korea; 6 weeks of age) were randomly assigned to the control, YM155, DOX, ESW + DOX, and YM155 + ESW + DOX groups (n = 8/group). As described previously, we generated a model of acute DOX-induced cardiomyopathy in mice using the following procedures. Mice in the DOX group received a single dose of DOX (15 mg/kg, i.p.)^35^. Control mice received injections of saline of comparable volume. Mice of YM155 and YM155 + ESW + DOX groups received injections of YM155 (3 mg/kg, i.p), followed by three times in 1 week for 14 days for boosting^29,31^. Mice assigned to the ESW + DOX and YM155 + ESW + DOX groups received *in vivo* ESW as described above; mice were subjected to 1,000 shots of ESW (0.04 mJ/mm^2^) 1 h before the DOX injection (15 mg/kg, i.p.), followed three times in 1 week for 14 days for boosting under inhalational anesthesia^22,23,52^. Fourteen days after the DOX injection, echocardiographic measurements were performed and the hearts were removed from the mice.

### Echocardiography

Echocardiography was performed using a commercially available HDI 5000 ultrasound scanner (Phillips Medical Systems, Bothell, WA, USA) with a 15 MHz linear array transducer. The animals were lightly anesthetized with 1–2% Terrell isoflurane during the echocardiographic examination. The M-mode images from the parasternal long-axis view were used to measure conventional echocardiographic parameters, including the left ventricular end diastolic dimension (LVEDD) and the left ventricular end systolic dimension (LVESD). LV function was assessed by fractional shortening (FS) and the ejection fraction (EF) was calculated from the LV linear measurements (LVEDD and LVESD).

### Western blotting

The cells were harvested in lysis buffer containing 1% protease and phosphatase inhibitors. After the lysates were centrifuged at 13,000 rpm for 30 min, the supernatants were collected. The protein concentrations of the cell lysates were measured using a BCA protein assay kit (Thermo Scientific, Waltham, MA, USA). Identical amounts of proteins were subjected to sodium dodecyl sulfate-polyacrylamide gel electrophoresis and transferred to nitrocellulose membranes. The membranes were blocked in 5% skim milk in TBST for 1 h and were incubated overnight with primary antibodies. The primary antibodies were used to detect the expression of survivin (1:1,000; Cat#2808, Cell Signaling Technology, Danvers, MA, USA), Bcl2 (1:500; Cat#sc-7382, Santa Cruz Biotechnology, Dallas, TX, USA), p-Akt (1:1,000; Cat#4060, Cell Signaling Technology), Akt (1:1,000; Cat#4685, Cell Signaling Technology), Sp1 (1:1,000; Cat#07-645, EMD Millipore Corp., Temecula, CA, USA), p53 (1:1,000; Cat#2524, Cell Signaling Technology), lamin A/C (1:1,000; Cat#sc-20681, Santa Cruz Biotechnology) and GAPDH (1:1,000; Cat#sc-25778, Santa Cruz Biotechnology). Total protein expression was normalized to GAPDH.

### Reverse transcription-polymerase chain reaction (RT-PCR)

Total cellular RNA was isolated from cultured H9c2 cells using a Total RNA Isolation Kit (Qiagen, Valencia, CA, USA) according to the manufacturer’s instructions. Complementary DNA (cDNA) was synthesized using M-MLV reverse transcriptase (Promega, Madison, WI, USA) and the oligo-dT 15 primer (Promega). cDNA was amplified with oligonucleotide primers during 30 PCR cycles. The following primer sequences were used: survivin primers 5′-ATGGGTGCTACGGCGCTGCCC-3′ and 5′-TCAGCGTAAGGCAGCCAGCTG-3′, GAPDH primers 5′-AGACAGCCGCATCTTCTTGT-3′ and 5′-CTTGCCGTGGGTAGAGTCAT-3′.

### Cell viability assay and terminal deoxynucleotidyl transferase dUTP nick-end labeling (TUNEL) assay

After the H9c2 cells were exposed to the indicated stimuli and incubated for 24 h, cell viability was measured using a cell viability assay kit (Abfrontier, Seoul, Korea) according to the manufacturer’s protocol. The TUNEL assay was performed using the DeadEnd Fluorometric TUNEL System (Promega) according to the manufacturer’s instructions. TUNEL-positive cells with green fluorescein were visualized with the LSM 800 instrument (Carl Zeiss, Oberkochen, Germany). To determine the percentage of apoptotic cells, the TUNEL-positive nuclei and TUNEL-negative cells were counted using the Image J software (National Institutes of Health).

### Small interference RNA (siRNA) transfection

To knockdown survivin expression, double strands of target small interference RNAs (siRNAs) were purchased from Bioneer (Daejeon, Korea). The following targeting sequences were used for survivin siRNA: 5′-GCA AAG GAG ACC AAC AAC AUU-3′ and 5′-UGU UGU UGG UCU CCU UUG CUU-3′. The transfection of siRNA was performed using Lipofectamine 3000 (Invitrogen) according to the manufacturer’s protocols. Twenty-four hours after siRNA transfection, the H9c2 cells were exposed to ESW followed by 1 μM of DOX treatment for 24 h.

### Nuclear/cytosol fractionation

The nuclear and cytosolic fractions were prepared using a nuclear/cytosol fractionation kit (Biovision, Milpitas, CA, USA) according to the manufacturer’s instructions. The purity of the protein fractions was assessed by immunoblotting the fractions with anti-lamin A/C (nuclear protein) and anti-GAPDH (cytoplasmic protein) antibodies.

### Immunofluorescence staining

The hearts of the mice were collected for immunofluorescence staining at 14 days after the DOX injection. Then the hearts, specifically the myocardial regions, were sectioned at 5 μm thickness. All animal procedures were approved by the Research Ethics Committees of Ewha Womans University and conducted in accordance with approved guidelines. After fixation in 4% paraformaldehyde (Biosesang Inc., Seongnam, Korea), the tissues were blocked in 10% normal goat serum and incubated with anti-mouse Bcl2 antibody (1:100; Santa Cruz Biotechnology) and anti-rabbit survivin antibody (1:400; Cell Signaling Technology) overnight at 4 °C. Anti-mouse AlexaFluor 647 goat (Life Technologies, Carlsbad, CA, USA) and anti-rabbit AlexaFluor 568 goat antibodies (Life Technologies) were used as the secondary antibodies. After nuclear counter-staining with DAPI, the tissue slides were mounted and visualized with the LSM 800.

### Statistical analysis

All of the data are expressed as means ± SEMs of at least three independent experiments. The nonparametric Mann-Whitney U-test was used for testing differences in quantitative variables. *p* values less than 0.05 were considered statistically significant.

## Data Availability

The datasets generated and/or analyzed during this study are included in this published article.

## Supplementary information

Supplementary Information
