# Supplementary material for: Extracorporeal shock waves protect cardiomyocytes from doxorubicin-induced cardiomyopathy by upregulating survivin via the integrin-ILK-Akt-Sp1/p53 axis
Source: Sci Rep. 2019 Aug 21;9:12149. doi: 10.1038/s41598-019-48470-0 (PMC6704172; doi:10.1038/s41598-019-48470-0)
Supplement: Supplementary file 1 — Supplementary Information [file 41598_2019_48470_MOESM1_ESM.docx]

**Supplementary Information**

**Extracorporeal shock waves protect cardiomyocytes from doxorubicin-induced cardiomyopathy by upregulating survivin via the integrin-ILK-Akt-Sp1/p53 axis**

Ji Yoon Lee^1^, Jihwa Chung^1^, Kyoung Hwa Kim^1^, Shung Hyun An^1^, Jeong-Eun Yi ^2^,

Kyoung Ae Kwon^3^ and Kihwan Kwon*^1, 2^

^1^ Medical Research Institute, School of Medicine, Ewha Womans University, Seoul, 158-710, Korea

^2^ Department of Internal Medicine, Cardiology Division, School of medicine, Ewha Womans University, Seoul, 158-710, Korea

^3^ Graduate School of Industrial Pharmaceutical Sciences, Ewha Womans University, Seoul, Korea

**Supplementary Figure**


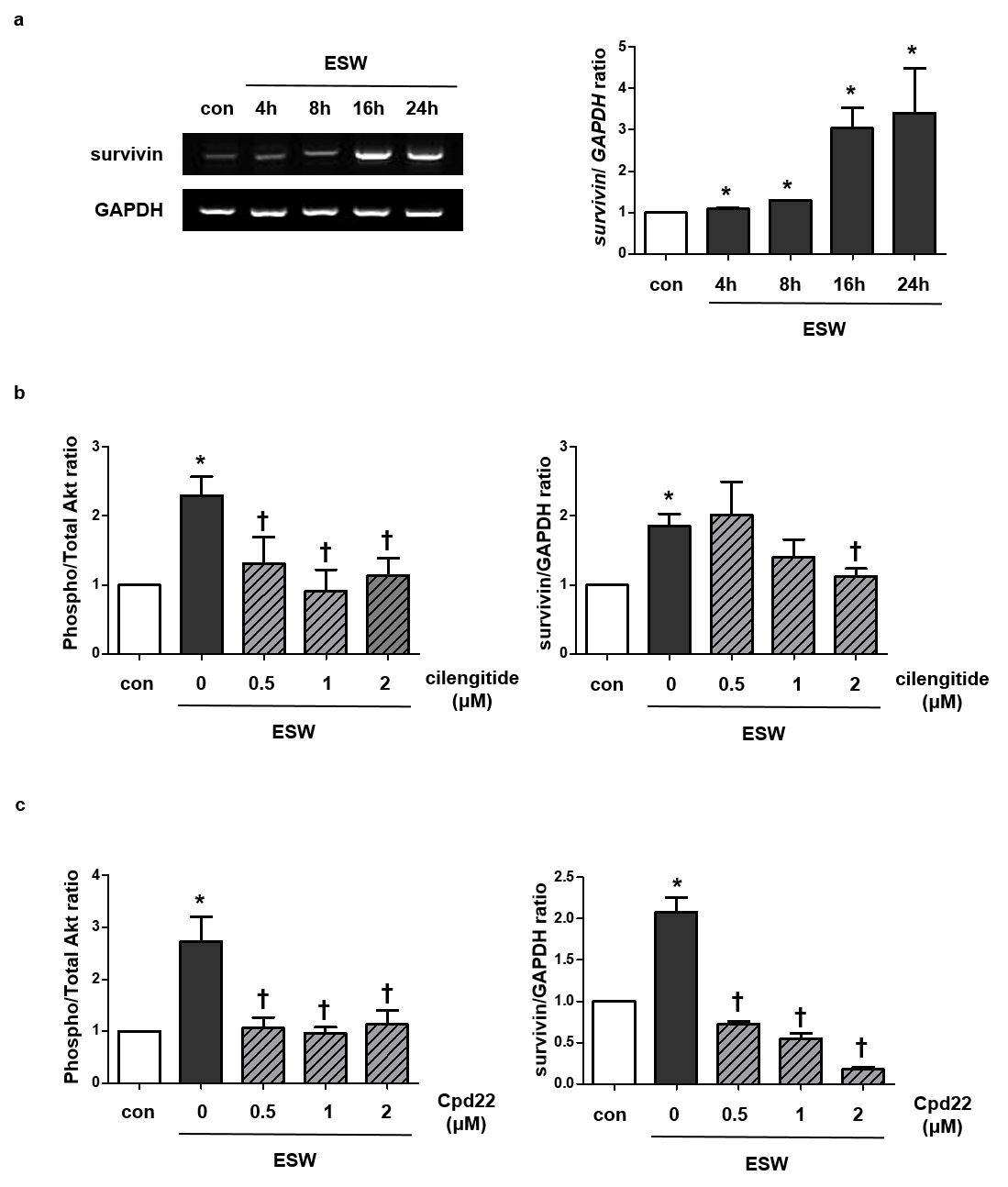


**Supplementary Figure S1. ESW upregulates survivin through the integrin/ILK/Akt signaling pathway in cardiomyocytes.**

**(a)** After being subjected to 1,000 shots of ESW (0.04 mJ/mm^2^$\mathrm{mm}^{2}$) at 4 Hz for 4 min, H9c2 cells were incubated in a 5% CO_2_ $\mathrm{CO}_{2}$incubator for the indicated time periods. The mRNA levels of *survivin* and *GAPDH* (internal control) were measured by reverse transcription-polymerase chain reaction (RT-PCR). Representative images are shown. **(b)** and **(c)** The cells were exposed to ESW after treatment with cilengitide or Cpd22 at the indicated concentrations for 2 h, or left untreated. The cells were harvested after a static incubation for 30 min or 24 h. The bar graphs shows the relative protein expression levels of phospho/total Akt and survivin, which are normalized to GAPDH (internal control) and indicated relative to those of the control. *Significant difference compared to control (*p* < 0.05). †Significant difference compared to DOX condition (*p* < 0.05).


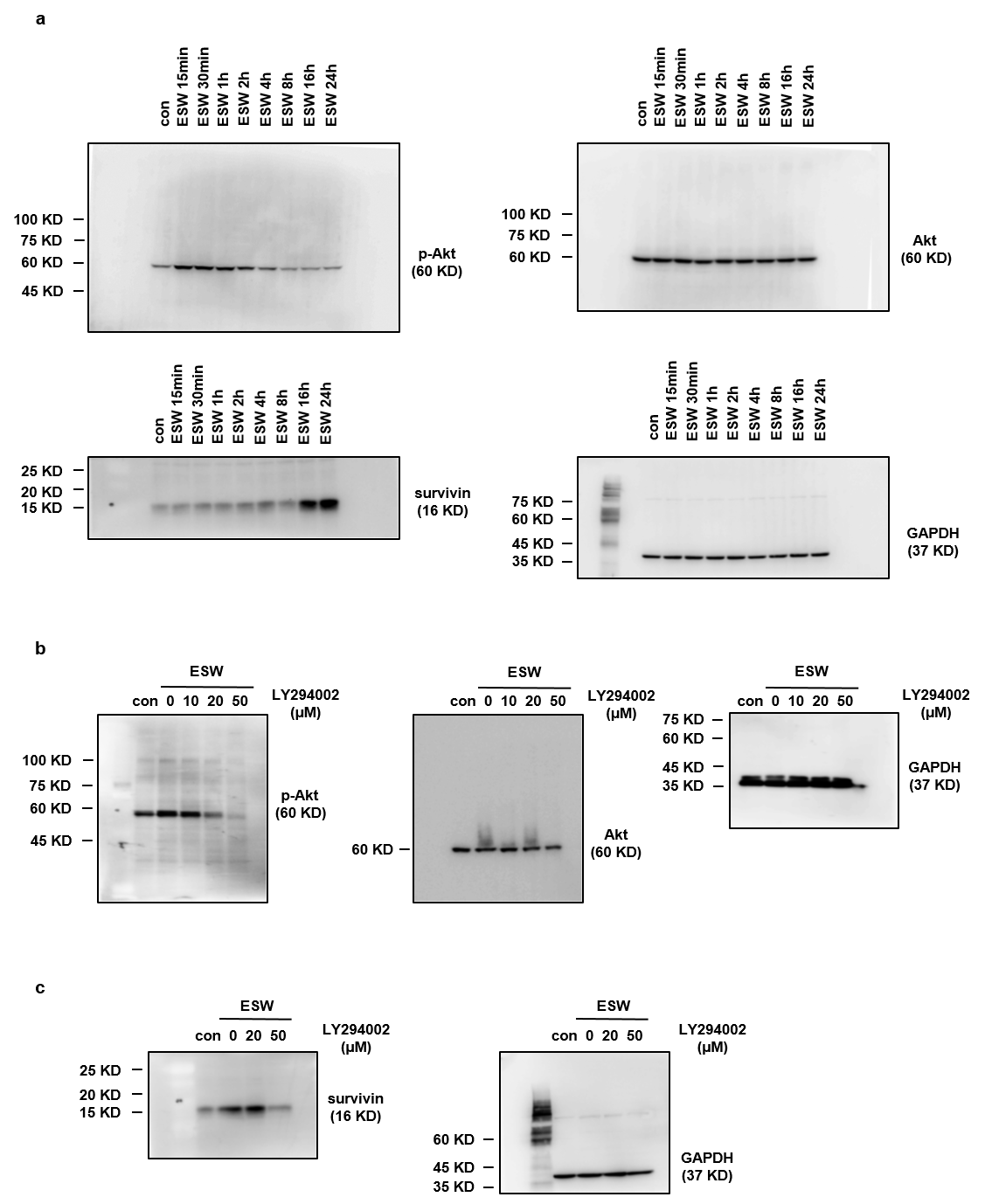


**Supplementary Figure S2. The full-length blots of Fig. 1a-c.**

**(a**) After treatment with 1,000 shots of ESW (0.04 mJ/mm^2^$\mathrm{mm}^{2}$), H9c2 cells were incubated in a 5% CO_2_$\mathrm{CO}_{2}$ incubator at 37°C for the indicated time periods. The protein levels of p-Akt, Akt, survivin, and GAPDH were measured by Western blot. **(b)** and **(c)** The cells were exposed to ESW after treatment with LY294002 at the indicated concentrations for 2 h or left untreated. The cells were harvested after a 30 min or 24 h incubation under a static condition. The protein expression levels of p-Akt, Akt, survivin, and GAPDH were measured by Western blot. The pictures near bands on membranes were cropped horizontally for Fig. 1a-c.


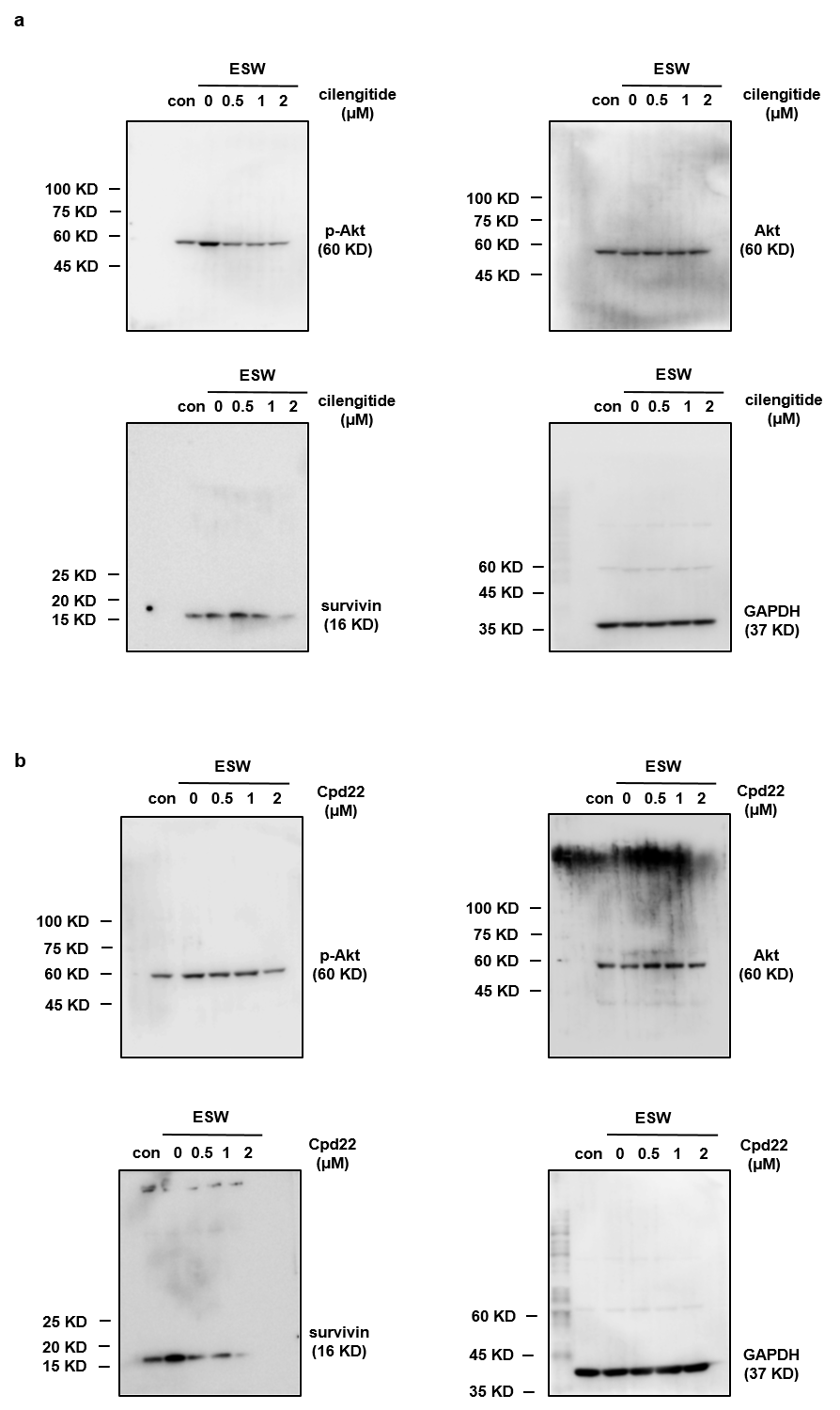


**Supplementary Figure S3. The full-length blots of Fig. 1d and 1e.**

**(a**) and **(b)** The cells were exposed to ESW after treatment with cilengitide or Cpd22 at the indicated concentrations for 2 h or left untreated. The cells were harvested after a 30 min or 24 h incubation under a static condition. The protein expression levels of p-Akt, Akt, survivin, and GAPDH were measured by Western blot. The pictures near bands on membranes were cropped horizontally for Fig. 1d and 1e.


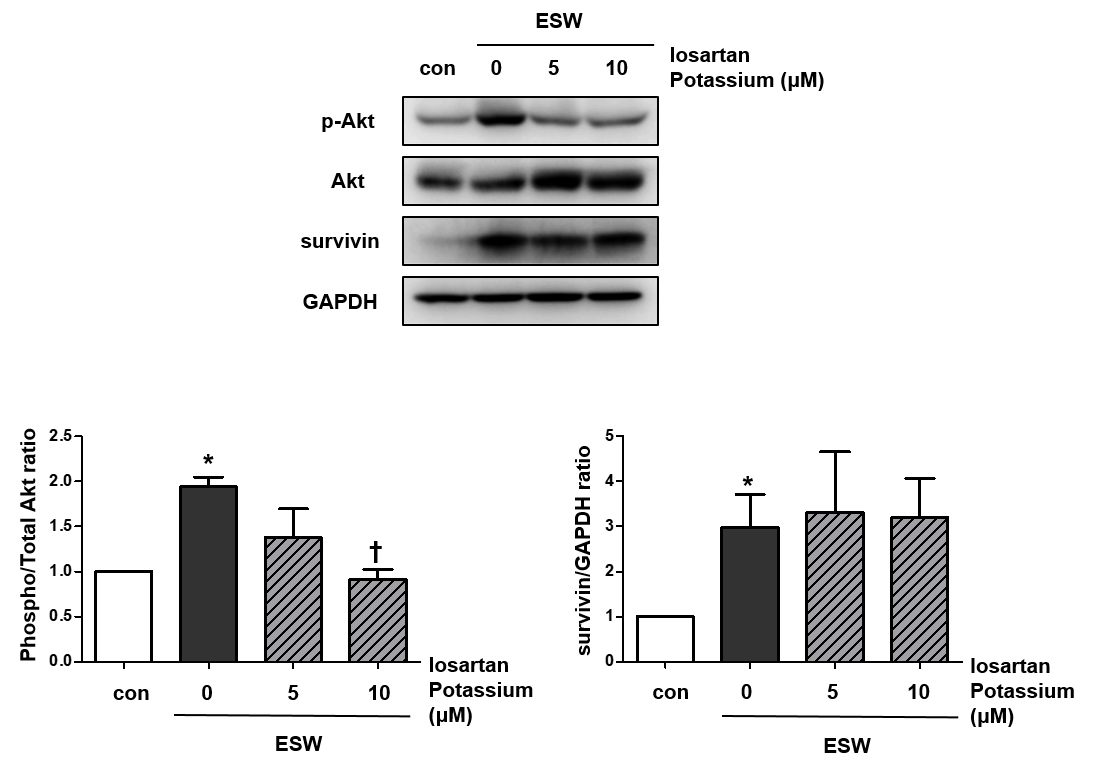


**Supplementary Figure S4. Inhibiting AT1R reduces phosphorylation of Akt induced by ESW, but not the expression of survivin**.

The cells were exposed to ESW after being treated with losartan potassium at the indicated concentrations for 2 h or left untreated. The cells were harvested after a 30 min or 24 h static incubation. The bar graphs show the relative protein expression levels of phospho/total Akt and survivin, which are normalized to GAPDH (internal control). The expression levels are indicated relative to those of the control. *Significant difference compared to the control (*p* < 0.05). †Significant difference compared to the DOX condition (*p* < 0.05).


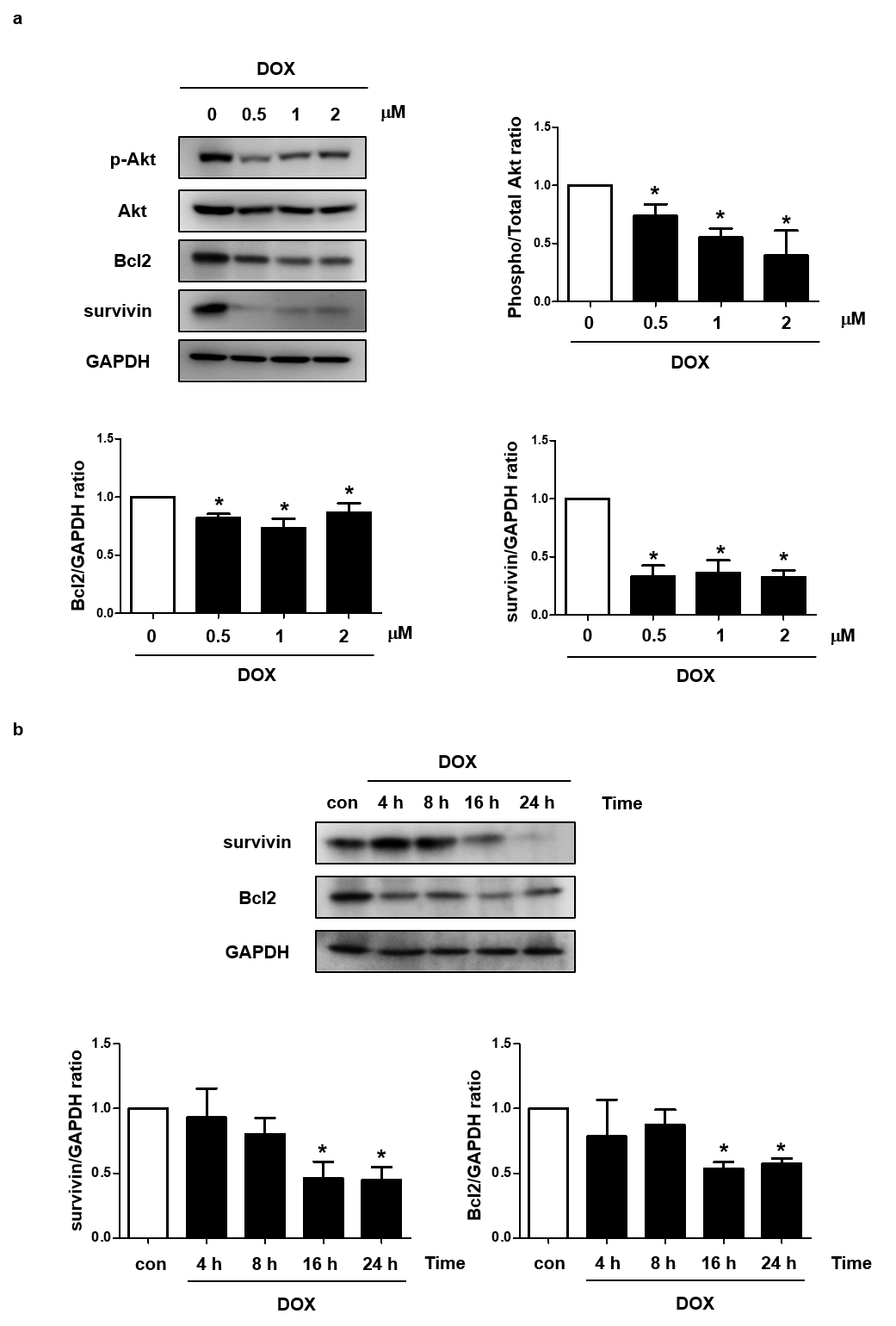


**Supplementary Figure S5. DOX treatment induces cellular apoptosis in dose/time-dependent manner by downregulating phosphorylation of Akt, survivin and Bcl2 in H9c2 cells.**

**(a**) H9c2 cells were left untreated or treated with DOX for the indicated dose for 24 h. (b) H9c2 cells were left untreated or treated with 1 μM for the indicated time periods. The protein expression levels of p-Akt, Akt, survivin, Bcl2, and GAPDH were measured by Western blot and normalized to GAPDH (internal control). The expression levels are indicated relative to those of the control. *Significant difference compared to control (*p* < 0.05).


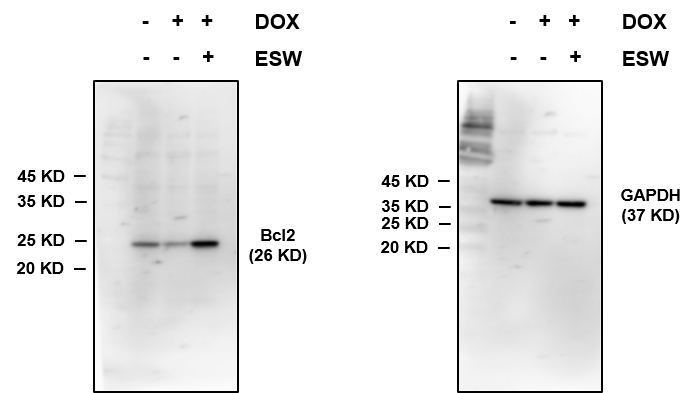


**Supplementary Figure S6. The full-length blots of Fig. 2d.**

H9c2 cells were left untreated or treated with 1,000 shots of ESW (0.04 mJ/mm^2^$\mathrm{mm}^{2}$), and then statically incubated in a 5% CO_2_ incubator for 1 h. After the incubation, cells were treated with 1 μM DOX for 24 h. The protein expression levels of Bcl2 and GAPDH were measured by Western blot. The pictures near bands on membranes were cropped horizontally for Fig. 2d


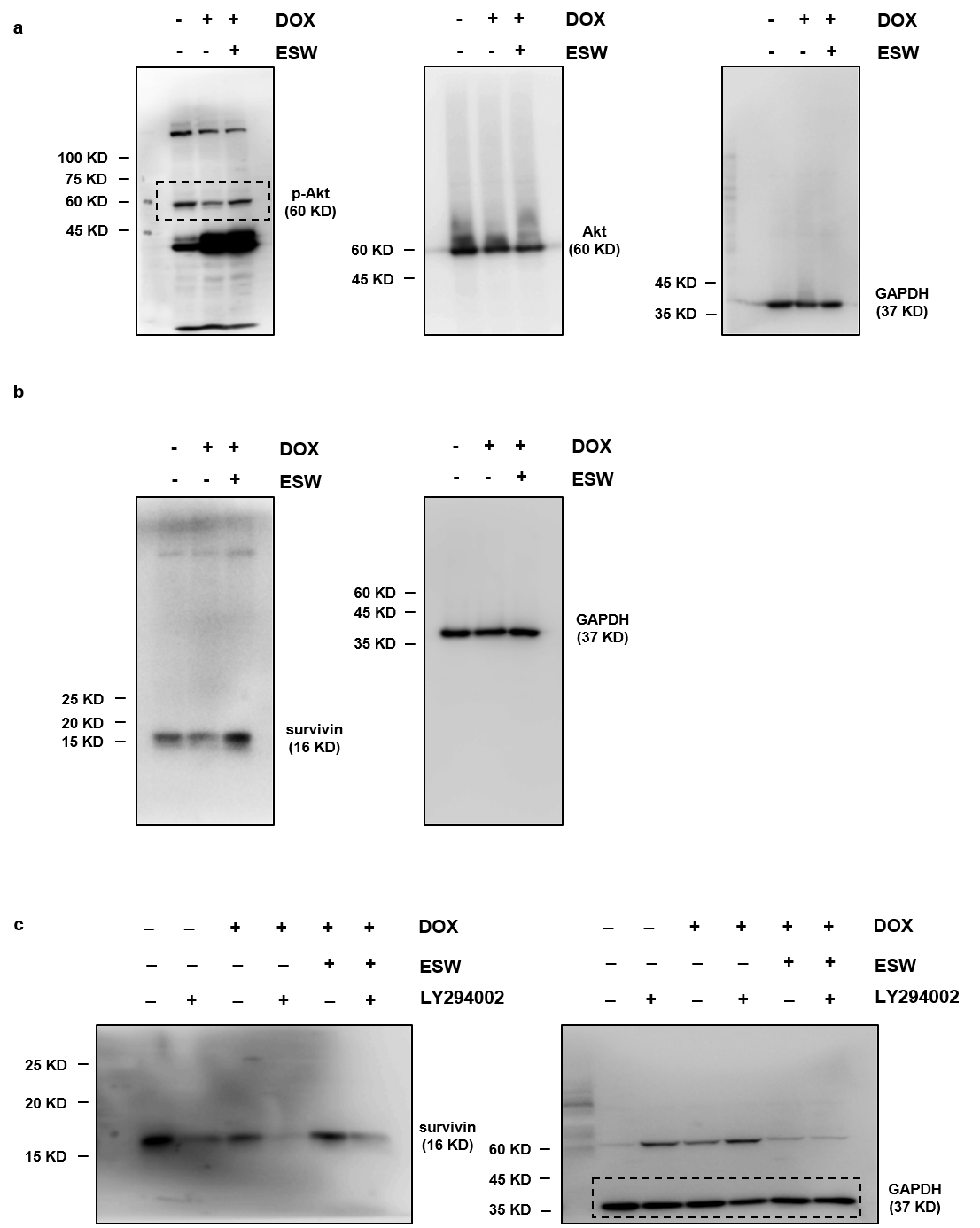


**Supplementary Figure S7. The full-length blots of Fig. 3a-c.**

**(a)** and (**b**) H9c2 cells were cultured in 1 μM DOX for 8 or 24 h 1 h after ESW. The protein expression levels of p-Akt, Akt, survivin, and GAPDH were measured by Western blot. **(c)** After treatment with 50 μM LY294002 for 2 h, the cells were exposed to ESW or left unexposed. After a 1 h incubation, the cells were treated with 1 μM DOX for 24 h. The protein expression levels of survivin and GAPDH were measured by Western blot. The main bands were marked with dotted boxes. The pictures near bands on membranes were cropped horizontally for Fig. 3a-c.


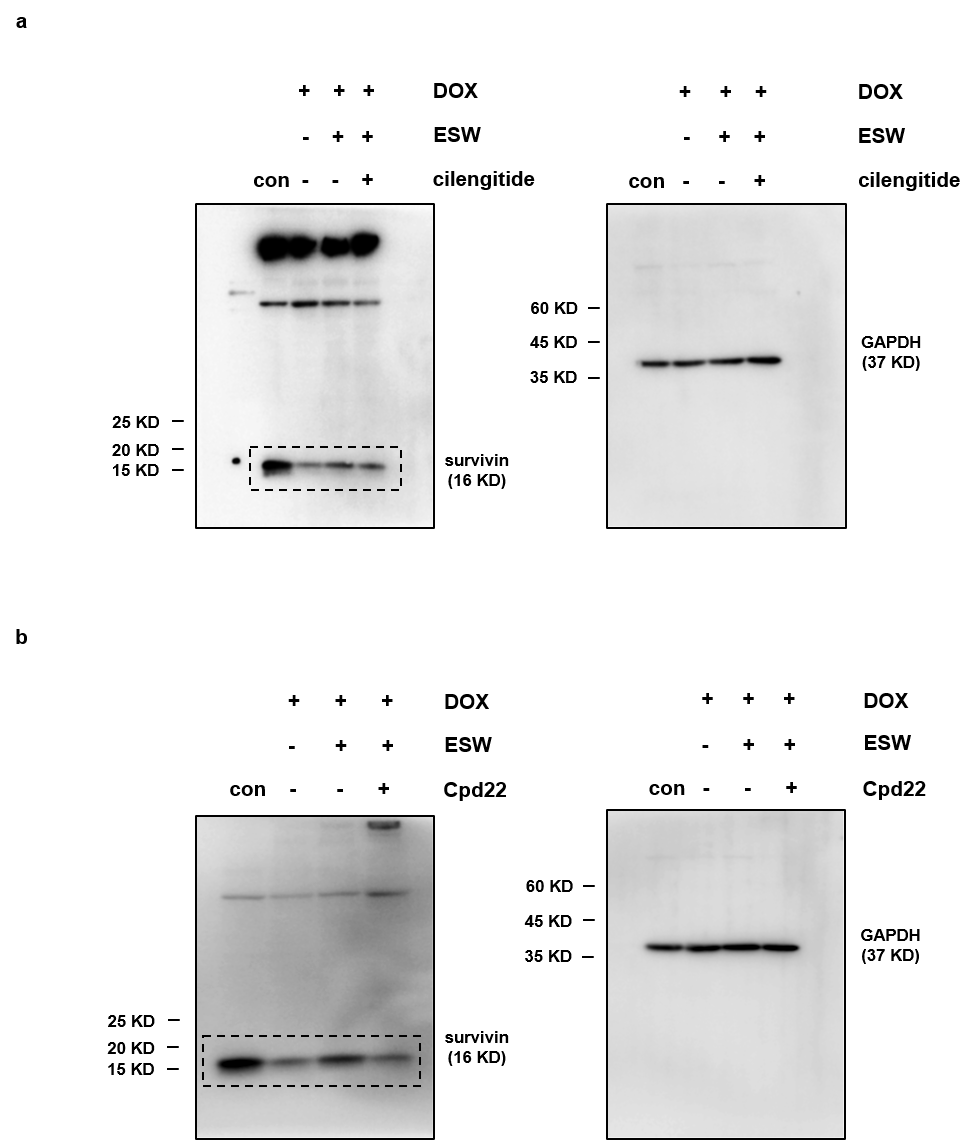


**Supplementary Figure S8. The full-length blots of Fig. 3d and 3e.**

**(a**) and **(b)** After treatment with 2 μM cilengitide or Cpd22 for 2 h, H9c2 cells were exposed to ESW or left unexposed. After a 1 h incubation, the cells were cultured in 1 μM DOX for 24 h. The protein expression levels of survivin and GAPDH were measured by Western. The main bands were marked with dotted boxes. The pictures near bands on membranes were cropped horizontally for Fig. 3d and 3e.

**
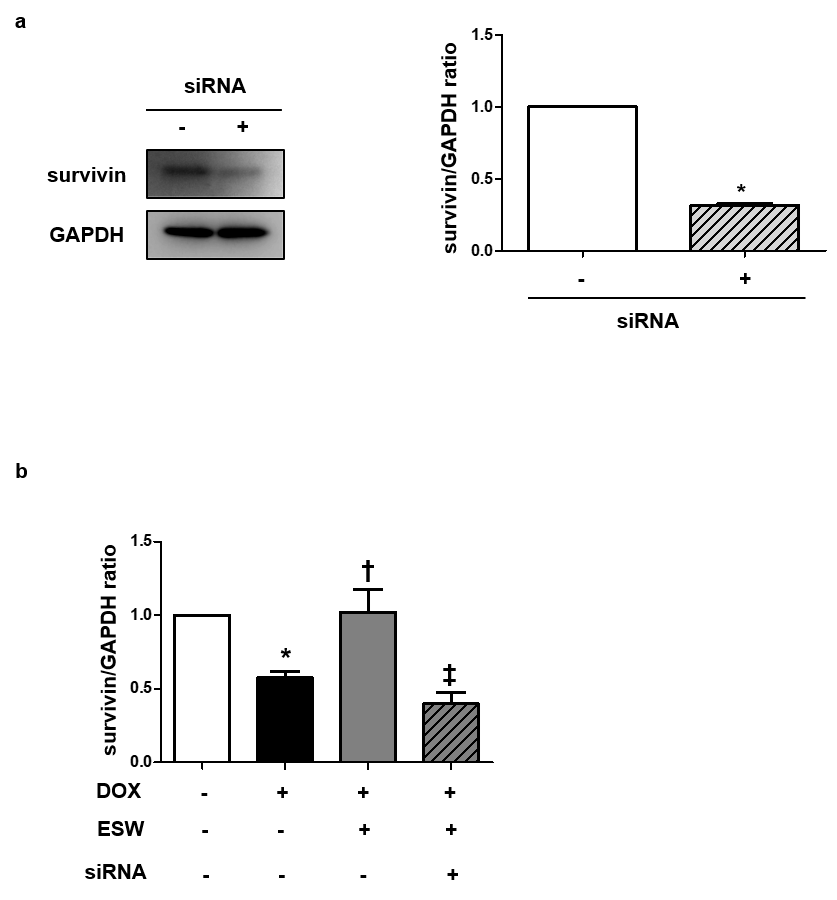
**

**Supplementary Figure S9. Transfection of siRNA targeting survivin efficiently reduced both endogenous and ESW-induced survivin expression (a)** H9c2 cells were treated with 50 nM siRNA targeting *survivin* for 24 h. **(b)** After treated with 50 nM siRNA for survivin for 24 h, H9c2 cells were subjected to ESW 1 h prior to the 1 μM DOX treatment. The cells were harvested after a 24 h incubation. The protein expression levels of survivin and GAPDH were measured by Western blot and normalized to GAPDH (internal control). The expression levels are indicated relative to those of the control. *Significant difference compared to control (*p* < 0.05). †Significant difference compared to the DOX condition (*p* < 0.05). ‡Significant difference compared to the ESW + DOX condition (*p* < 0.05).

**
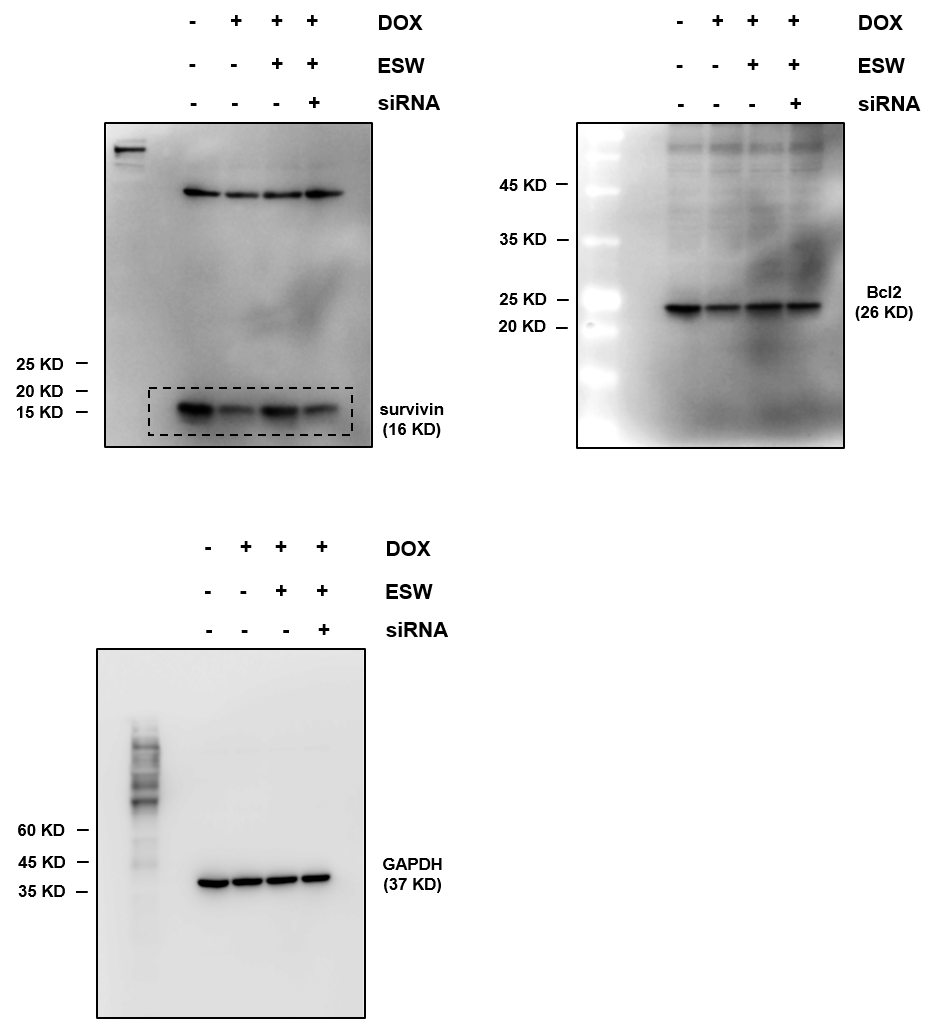
**

**Supplementary Figure S10. The full-length blots of Fig. 4a.**

After siRNA transfection targeting *survivin* for 24 h, H9c2 cells were subjected to ESW 1 h prior to the DOX treatment. The cells were harvested after a 24 h incubation. The protein expression levels of survivin, Bcl2, and GAPDH were measured by Western blot. The main bands were marked with dotted boxes. The pictures near bands on membranes were cropped horizontally for Fig. 4a.

**
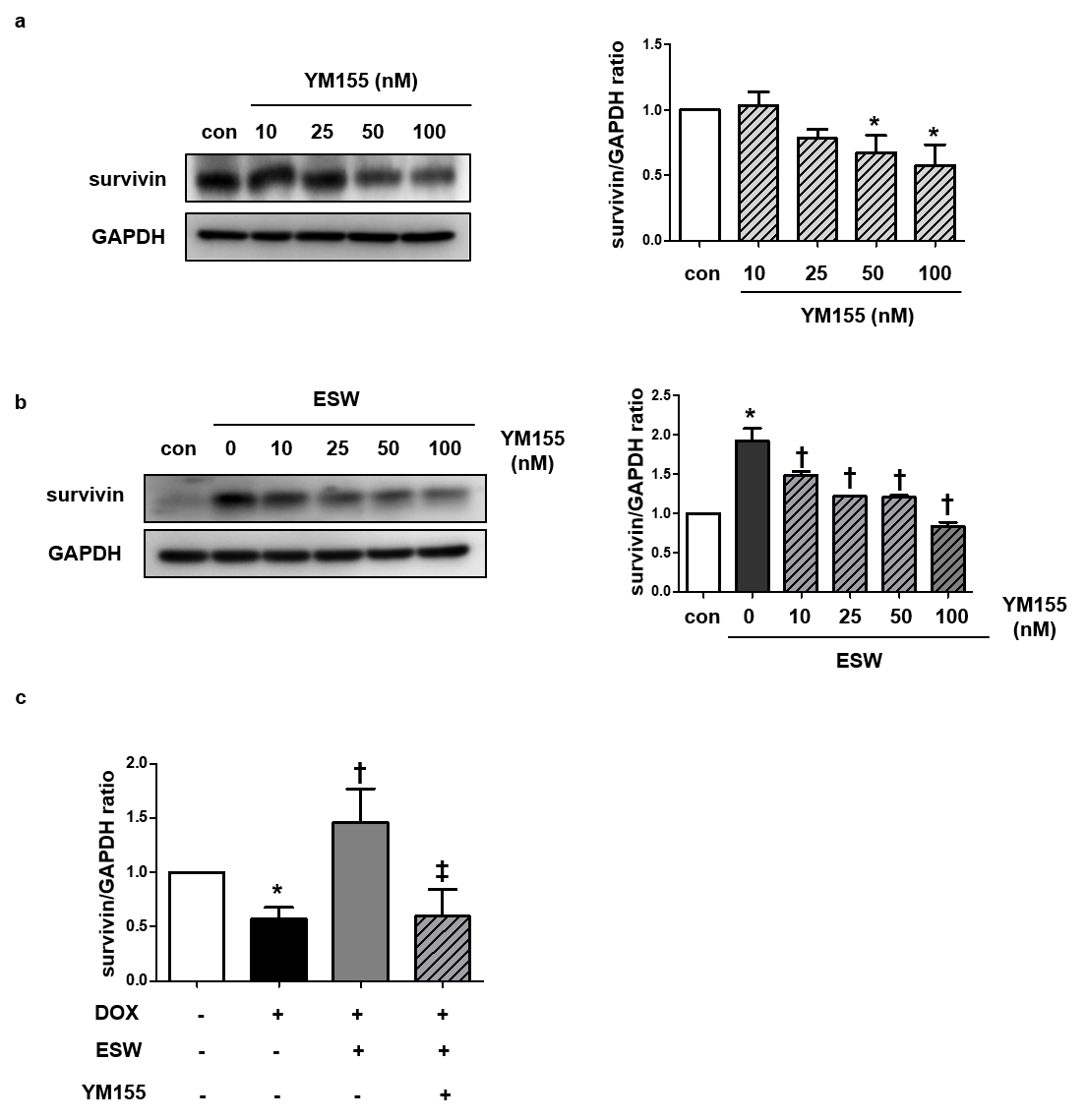
**

**Supplementary Figure S11. YM155 inhibits endogenous and ESW-induced survivin expression.**

**(a)** H9c2 cells were incubated for 24 h after being treated with YM155 at the indicated concentrations. **(b)** H9c2 cells were subjected to ESW after being treated with YM155 at the indicated concentrations for 3 h. The cells were harvested after a 24 h static incubation. The protein expression levels of survivin and GAPDH were measured by Western blot and normalized to GAPDH (internal control). The expression levels are indicated relative to those of the control. **(c)** The cells were exposed to ESW after being treated with 100 nM YM155 for 3 h or left untreated. The cells were harvested after a 24 h incubation under 1 μM DOX condition. The bar graphs show the relative protein expression levels of survivin, which are normalized to GAPDH (internal control) and indicated relative to those of the control. *Significant difference compared to control (*p* < 0.05). †Significant difference compared to the DOX condition (*p* < 0.05). ‡Significant difference compared to the ESW + DOX condition (*p* < 0.05).

**
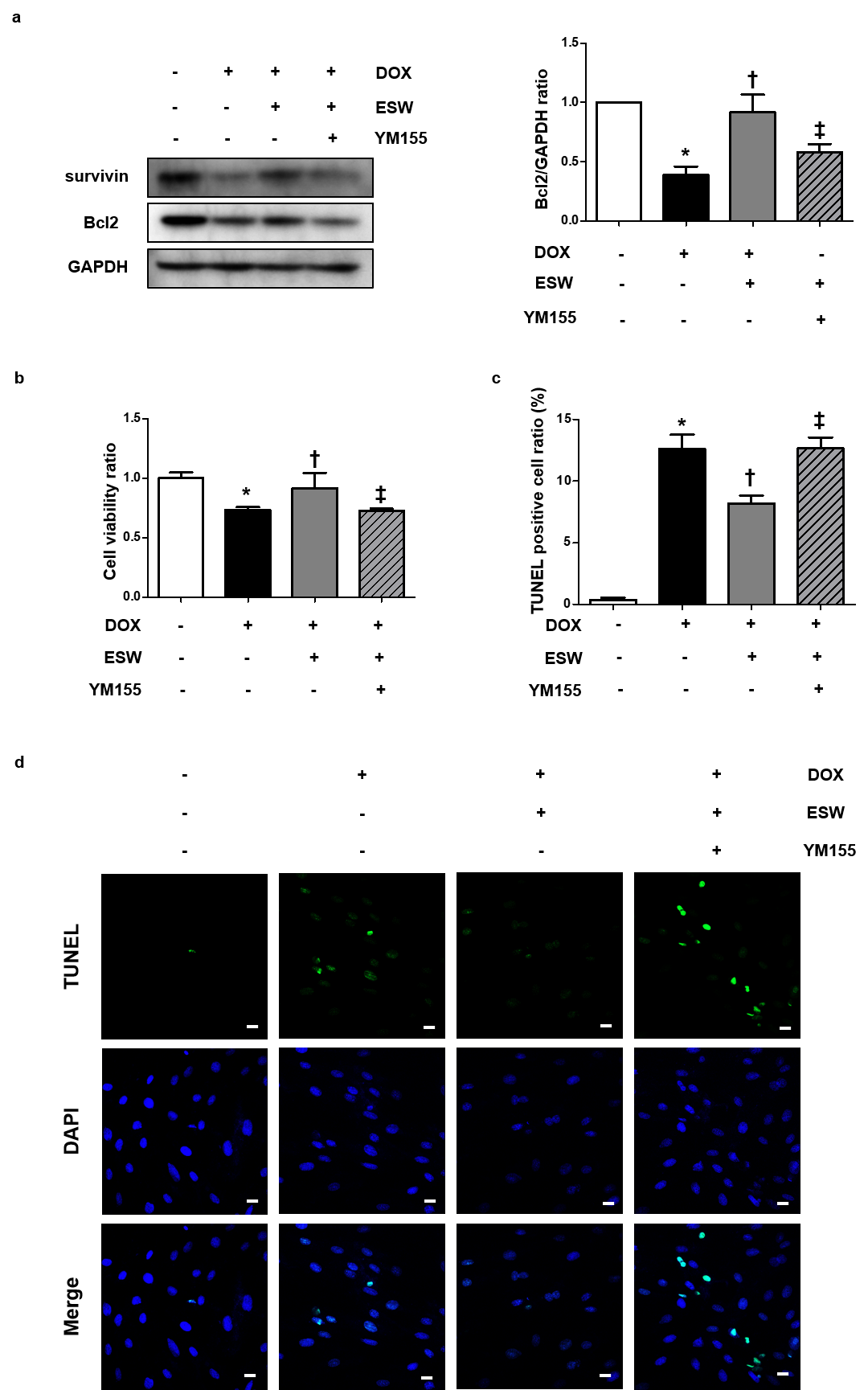
**

**Supplementary Figure S12. Inhibition of survivin by YM155 alleviates the cardioprotective effect of ESW in DOX-induced cell death.**

After treating with 100 nM YM155 for 3 h, H9c2 cells were subjected to ESW 1 h prior to the DOX treatment. The cells were harvested after a 24 h incubation. **(a)** The protein expression levels of survivin, Bcl2, and GAPDH were measured by Western blot and normalized to GAPDH (internal control). The expression levels are indicated relative to those of the control. The full-length blots are presented in Supplementary Fig. S10. **(b)** Cell viability of each condition was measured by the MTT assay. The values for all conditions are indicated relative to those of the control. **(c)** The bar graph shows the TUNEL-positive cell ratio (%) as the cellular apoptotic index. The TUNEL-stained cells were counted and normalized to DAPI-stained cells: control (0.3703 ± 0.1718, means ± standard error), DOX (12.61 ± 1.159, means ± standard error) and ESW+DOX (8.194 ± 1.562, means ± standard error), YM155+ESW+DOX (12.65 ± 0.9013, means ± standard error). n=5, Mean ± SEM. *Significant difference compared to control (*p* < 0.05). †Significant difference compared to the DOX condition (*p* < 0.05) ‡Significant difference compared to the ESW + DOX condition (*p* < 0.05). **(d)** Representative fluorescence microscopic TUNEL images from at least three independent experiments are shown (magnification, 200×; scale bars, 10 μm).

**
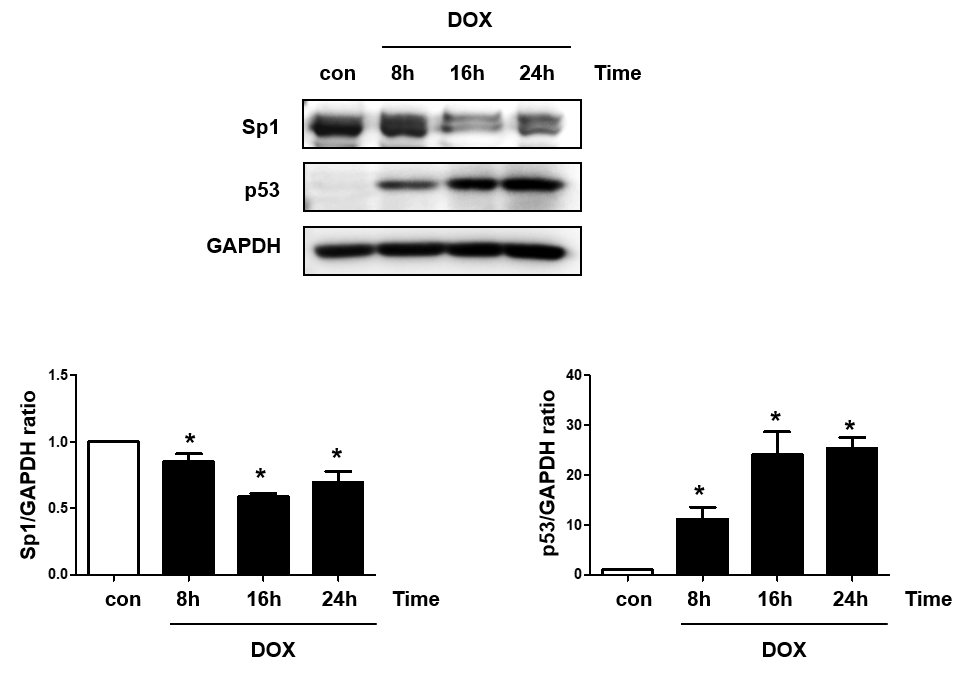
**

**Supplementary Figure S13. DOX regulates the expression of Sp1 and p53 in cardiomyocytes.**

The cells were harvested after being treated with 1 μM DOX for the indicated time periods. The protein expression levels of Sp1, p53, and GAPDH were measured by Western blot and normalized to GAPDH (internal control). The expression levels are indicated relative to those of the control. *Significant difference compared to control (*p* < 0.05).


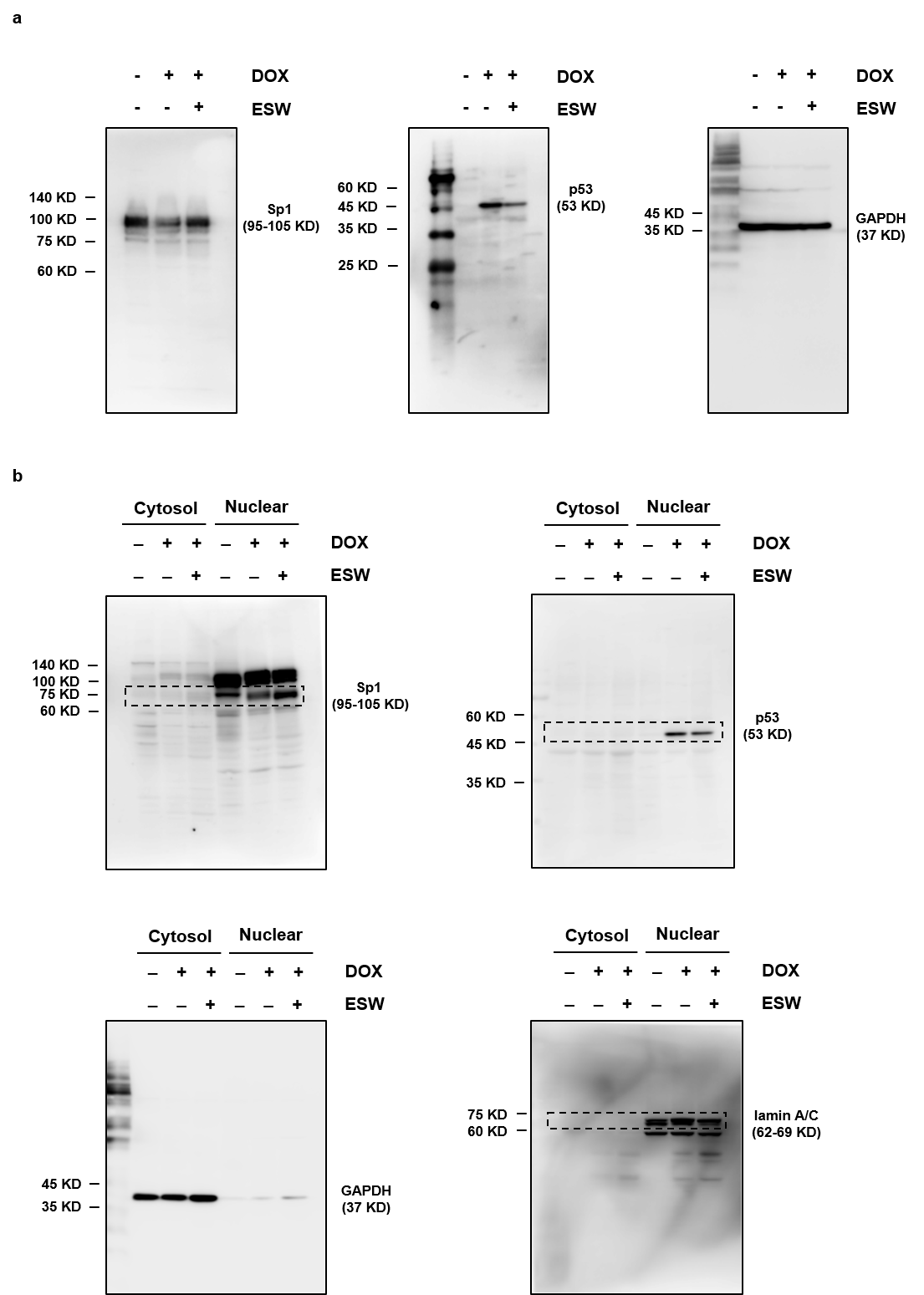


**Supplementary Figure S14. The full-length blots of Fig. 5a and 5b.**

The cells were cultured in 1 μM DOX for 24 h 1 h after being subjected or not to ESW. **(a)** The protein expression levels of Sp1, p53, and GAPDH were measured by Western blot. **(b)** Cytoplasmic and nuclear extracts isolated from H9c2 cells under each condition were separated by Western blot with antibodies against Sp1, p53, GAPDH, and lamin A/C. The main bands were marked with dotted boxes. The pictures near bands on membranes were cropped horizontally for Fig. 5a and 5b.


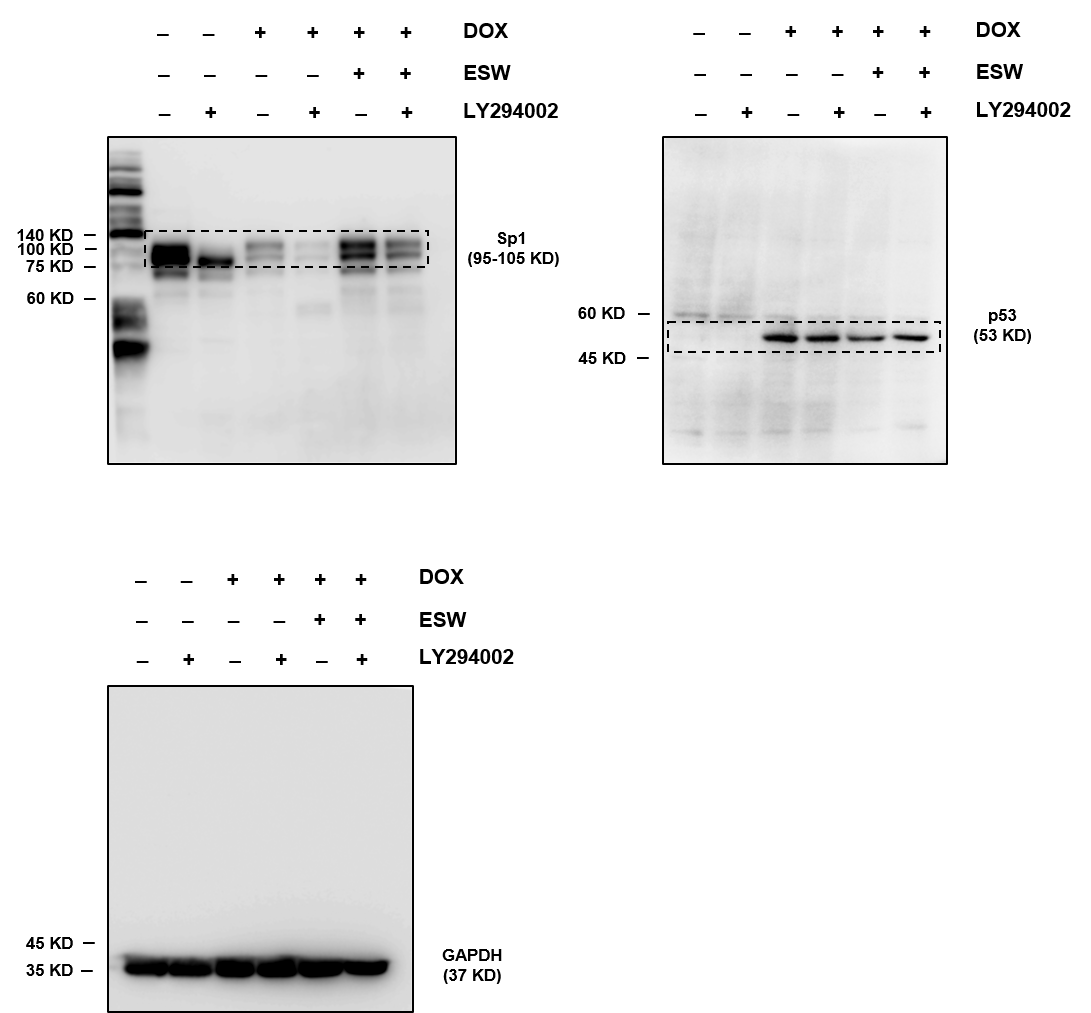


**Supplementary Figure S15. The full-length blots of Fig. 5c.**

After treatment with 50 μM LY294002 for 2 h, the cells were exposed to ESW or left unexposed. After a 1 h incubation, the cells were treated with 1 μM DOX for 24 h. The protein expression levels of Sp1, p53, and GAPDH were measured by Western blot. The main bands were marked with dotted boxes. The pictures near bands on membranes were cropped horizontally for Fig. 5a and 5b.


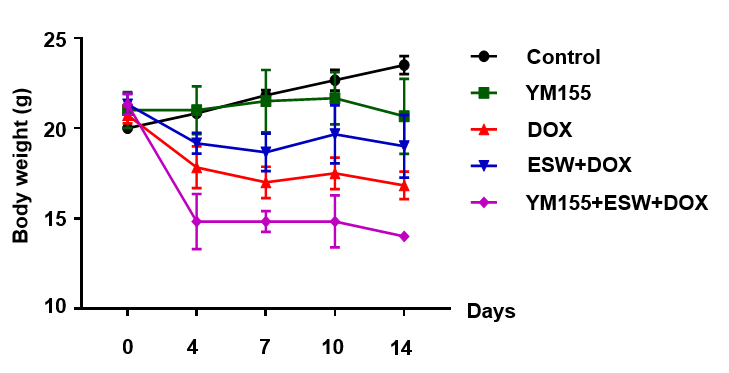


**Supplementary Figure S16. The body weight of mice in an *in vivo* mouse model during experimental periods.**

The body weight of mice assigned to control, YM155, DOX, ESW + DOX, and YM155 + ESW + DOX groups in an *in vivo* mouse model were measured 5 times during 14 days (n=8/group); day 0 indicates the day DOX, ESW + DOX and YM155 + ESW +DOX group received DOX (15 mg/kg, i.p) injection. The measurement of body weight of each mouse was repeated at least three times and the mean value was used in data.


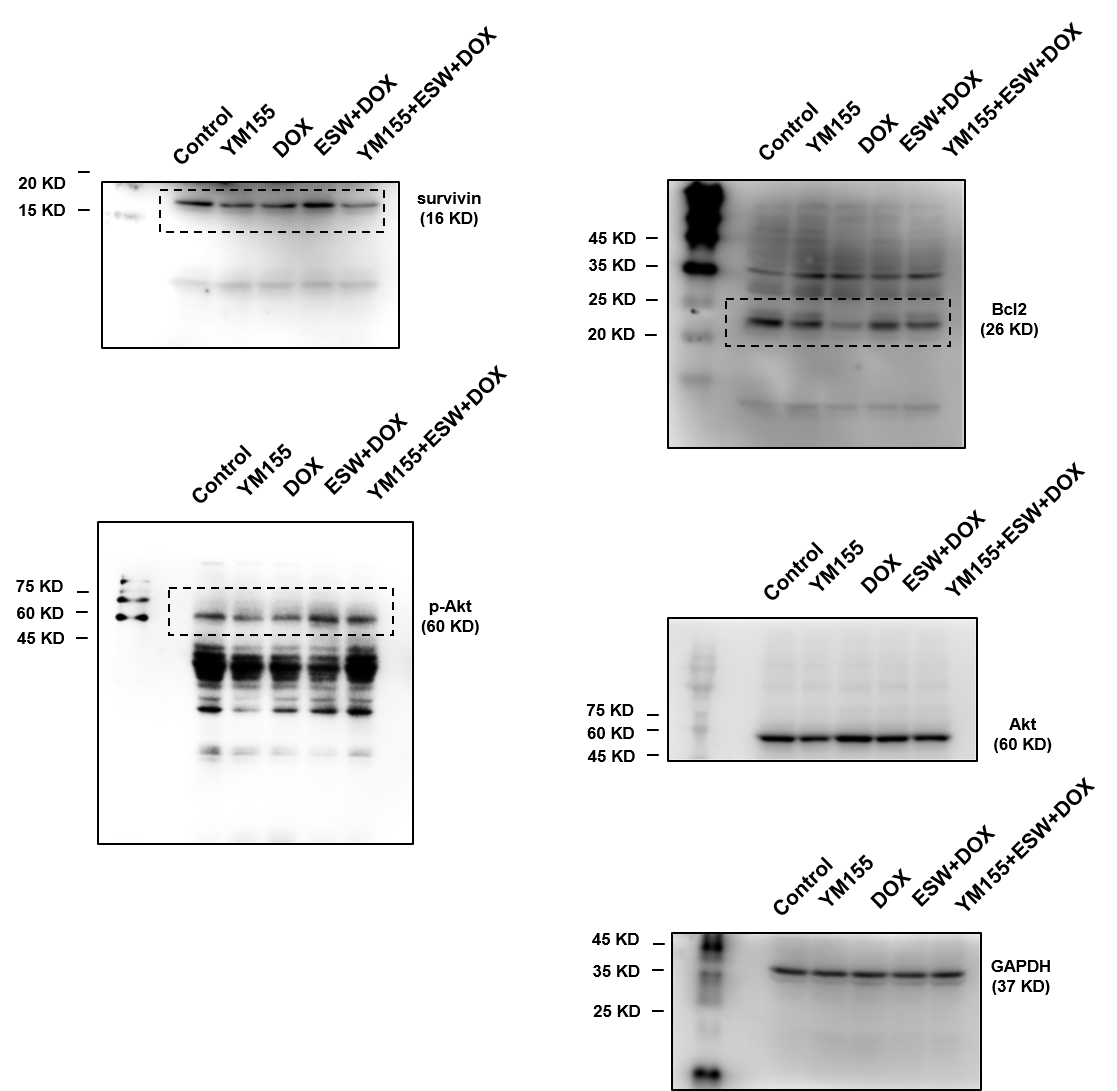


**Supplementary Figure S17. The full-length blots of Fig. 7b.**

Mice were assigned to control, YM155, DOX, ESW + DOX, and YM155 + ESW + DOX. The hearts of mice were harvested at 14 days after the DOX injection and examined by Western blot for p-Akt, Akt, survivin, Bcl2, and GAPDH. The main bands were marked with dotted boxes. The pictures near bands on membranes were cropped horizontally for Fig. 7b.


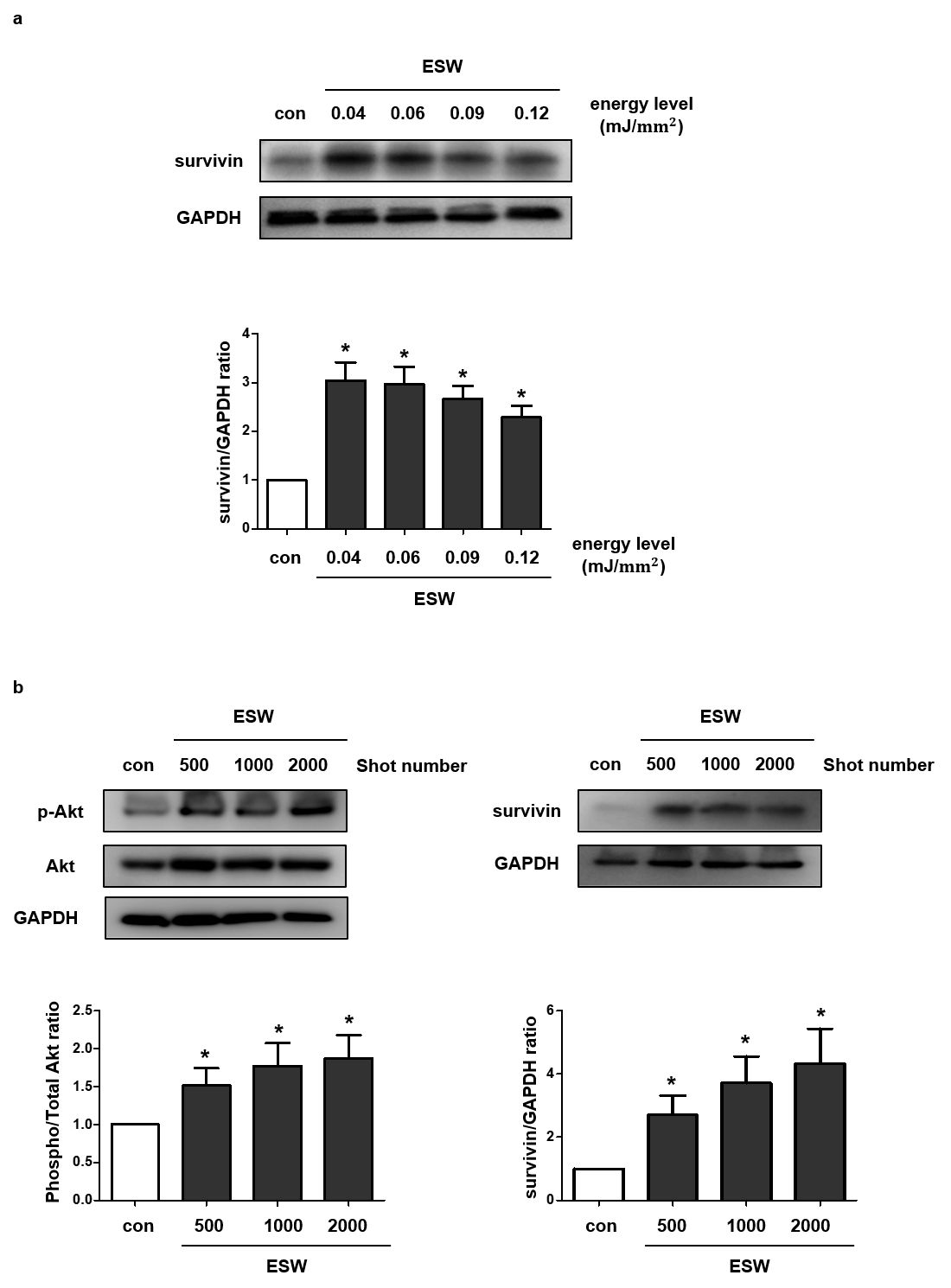


**Supplementary Figure S18. Effect of ESW treatment at different energy levels and shot numbers on Akt phosphorylation and survivin expression in cardiomyocytes.**

(a) H9c2 cells were exposed to 1,000 shots of ESW at different energy levels (0.04, 0.06, 0.09 and 0.12 mJ/mm^2^). (b) H9c2 cells were exposed to ESW (0.04 mJ/mm^2^) at different shot numbers (500, 1000, 2000 shots). The cells were harvested after 24 h static incubation. The bar graphs show the relative protein expression levels of Phospho/Total Akt ratio and survivin, which are normalized to GAPDH (internal control). The expression levels are indicated relative to those of the control. *Significant difference compared to the control (*p* < 0.05).

**Supplementary Tables**


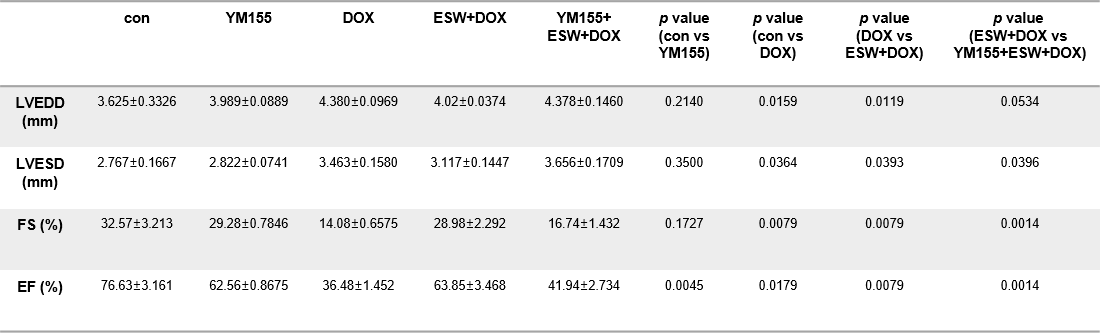


**Supplementary Table S1. ESW improves both dilated cardiomyopathy and cardiac functions induced by DOX, while inhibition of survivin attenuates its effect in an *in viv*o mouse model.**

Echocardiography was performed in the control, YM155, DOX, ESW + DOX, and YM155 + ESW + DOX groups at 14 days after the DOX injection. Quantitative echocardiographic group data: LVEDD (mm), LVESD (mm), fractional shortening (FS) (%) and ejection fraction (EF) (%) (n = 8/group). Values are means ± standard error. Each *p*-value is the result of a comparison between two groups (control vs. DOX, DOX vs. ESW + DOX, and ESW + DOX vs YM155 + ESW + DOX).
